# Continuum beliefs and mental illness stigma: a systematic review and meta-analysis of correlation and intervention studies

**DOI:** 10.1017/S0033291721000854

**Published:** 2021-04

**Authors:** Lina-Jolien Peter, Stephanie Schindler, Christian Sander, Silke Schmidt, Holger Muehlan, Thomas McLaren, Samuel Tomczyk, Sven Speerforck, Georg Schomerus

**Affiliations:** 1 Leipzig University, Medical Faculty, Department of Psychiatry and Psychotherapy, Leipzig, Germany; 2 University of Leipzig Medical Center, Department of Psychiatry and Psychotherapy, Leipzig, Germany; 3 University of Greifswald, Institute of Psychology, Department of Health and Prevention, Greifswald, Germany

**Keywords:** continuum beliefs, continuum model, mental health, meta-analysis, social distance, stigma, systematic review

## Abstract

**Background:**

Promulgating a continuum model of mental health and mental illness has been proposed as a way to reduce stigma by decreasing notions of differentness. This systematic review and meta-analysis examines whether continuum beliefs are associated with lower stigma, and whether continuum interventions reduce stigma.

**Methods:**

Following a pre-defined protocol (PROSPERO: CRD42019123606), we searched three electronic databases (PubMed, Web of Science, and PsycINFO) yielding 6726 studies. After screening, we included 33 studies covering continuum beliefs, mental illness, and stigma. Of these, 13 studies were included in meta-analysis.

**Results:**

Continuum beliefs are consistently associated with lower stigma. Interventions were effective at manipulating continuum beliefs but differ in their effects on stigmatising attitudes.

**Conclusions:**

We discuss whether and to what extent attitudes towards people with mental illness can be improved by providing information on a mental health-mental illness continuum. It appeared to be relevant whether interventions promoted a feeling of ‘us’ and a process of identification with the person with mental illness. We discuss implications for the design of future interventions.

## Introduction

The stigma of mental illness still affects the lives of most people with mental illness (Lasalvia et al., 2013; Thornicroft, Brohan, Rose, Sartorius, & Leese, 2009). Over the last decade, the concept of an underlying continuum of mental illness and mental health has inspired numerous studies examining whether continuum beliefs can reduce mental illness stigma. Assuming a continuum from mental health to illness is coherent with current social and biological understanding (Goldberg, 2000) and epidemiological findings. It corresponds to the dimensional classification of symptom severity in research criteria (ROC) and the use of thresholds of severity, frequency, and number of symptoms in current classifications such as DSM-5 and ICD-10. Similar to these classifications, for the purpose of this review, we define the term ‘mental illness’ broadly, including personality and substance use disorders (SUDs; Freeman, 2005).

A continuum concept of mental health and mental illness assumes one dimension from severe psychiatric symptoms to subclinical, light, or non-existent symptoms. Since every person is likely experiencing symptoms of mental illness at some points during their life, a person with mental illness might be seen as someone with similar, but more severe experiences, thus remaining ‘someone like us’. Continuum beliefs are framing how people perceive mental illness in general, they imply that someone's mental illness is not categorically distinct from normal behaviour but falls on a continuum of life experiences. Assumingly, continuum beliefs are amenable to interventions.

The opposite is a binary view of either mental health or mental illness, where people perceive the experience of mental illness to be fundamentally different from normal experiences and behaviour. Such categorical distinctions are, at a conceptual level, closely linked to the stigma process (Link & Phelan, 2001; Tajfel & Turner, 1979; Turner & Oakes, 1986), which begins by creating groups and labelling them. Negative stereotypes are linked to labels, leading to separation of ‘us’ from ‘them’, status loss, and discrimination (Link & Phelan, 2001). Mental illness stigma increases symptom burden, poses a barrier to help-seeking and fosters treatment avoidance (Angermeyer, van der Auwera, Carta, & Schomerus, 2017; Clement et al., 2015; Eisenberg, Downs, Golberstein, & Zivin, 2009; Henderson, Evans-Lacko, & Thornicroft, 2013; Schulze et al., 2020). Since underlying categorical beliefs are central to the stigma process, continuum beliefs can be regarded as a counter of stigma at a conceptual level.

Literature on continuum beliefs and stigma has multiplied over the last few years. This systematic review and meta-analysis summarises correlation and intervention studies to establish whether continuum beliefs are in fact associated with less stigma, and whether continuum beliefs should be used for the de-stigmatisation of mental illness. We particularly want to find out:

(1) How are continuum beliefs and stigmatising attitudes towards people with mental illness associated? Are there differences in associations depending on the type of mental illness?
(2) Are continuum beliefs amenable to interventions? Are changes in continuum beliefs accompanied by changes in stigmatising attitudes? Do effects differ between types of mental illness?

## Methodology

The study protocol was registered at PROSPERO as CRD42019123606 (available at https://www.crd.york.ac.uk/prospero/display_record.php?ID=CRD42019123606). The literature review was conducted following PRISMA guidelines (Moher, Liberati, Tetzlaff, & Altman, 2009).

### Search strategy

A title, abstract, and keyword search combined the main topics: continuum beliefs, stigma, mental health, and illness using Boolean operators. For mental illness, the search contained both general terms (e.g. ‘mental disorder*’) and prevalent disorders (e.g. depress* OR alcohol*). Search strategy and topics have been reviewed using the PRESS checklist (McGowan et al., 2016). Online Supplementary Appendix 1 contains search string and PICO questions.

The main search was conducted in January 2020 using PubMed, Web of Science, and PsycINFO with weekly e-mail alerts. Additional searches comprised of author correspondence and checking references lists. All eligible articles published before May 2020 were imported to Citavi 6 (Swiss Academic Software GmbH), automatically and manually removing any duplicates. The selection of articles was performed in a sequential manner based on pre-defined eligibility criteria (see below). First, articles dealing clearly with topics outside the scope of this review were eliminated through title screening (LJP, JK, and TG), followed by abstract (LJP) and full text screening (LJP and CS). In the screening stages of our review, we followed a liberal, over-inclusive approach, resolving all conflicts by consensus among four authors (LJP, CS, SS, and GS).

### Eligibility criteria

Included articles had to be related to (1) continuum beliefs of mental health and mental illness (in general, or specific mental-, substance use-, or personality disorders) and (2) stigmatisation of people with mental illness (including attitudes, emotional reactions, or stereotypes). In addition to not meeting (1) and (2), the following exclusion criteria applied: (3) not containing primary data (e.g. reviews, comments, etc.); (4) unpublished articles; (5) not referring to human samples or (6) language other than English, German or French. Measures employed by the retrieved studies are described below (‘Results’ section).

### Quality assessment

We assessed methodological quality using a modified version (see online Supplementary Appendix 2) of a 27 item-checklist originally developed to evaluate clinical intervention studies (Downs & Black, 1998) to allow a comparable risk of bias assessment. Two raters (LJP and CS) performed quality assessment independently; deviations were discussed with a third rater (SS).

### Data extraction and coding

A coding protocol was pre-defined (Schewe, Hülsheger, & Maier, 2014) containing study-, sample-, and effect size-level (see online Supplementary Appendix 1).

### Analytic strategy

Several studies with similar predictor and outcome measures showed sufficient homogeneity to be aggregated into meta-analysis, which was the case for associations of continuum beliefs and social distance, emotional reactions (pro-social reaction, fear, and anger), and stereotypes (dangerousness, unpredictability, and responsibility). Results regarding interventions were only addressed narratively.

Meta-analyses were conducted using Meta-Essentials 1.5 (Suurmond, van Rhee, & Hak, 2017). To be eligible for meta-analysis, studies needed to report either Pearson's correlation coefficients (*r*), unstandardised, or standardised (*β*) regression coefficients concerning the association of continuum beliefs and stigma-related outcomes. Due to differences between instruments, scale ranges, and study populations, random effects models were conducted (Riley, Higgins, & Deeks, 2011) with two-sided *p* values and 95% confidence intervals (CIs). Heterogeneity was estimated via *I*^2^ statistic, considering 25, 50, and 75% as thresholds of low, moderate, and high heterogeneity, respectively (Higgins, Thompson, Deeks, & Altman, 2003; Huedo-Medina, Sánchez-Meca, Marín-Martínez, & Botella, 2006).

We used a widespread meta-analytic approach by Peterson and Brown (2005), which suggests using *r* as effect-size and imputing missing *r*'s using *β* (analyses of social distance, pro-social reaction, fear, and anger). Further analyses were undertaken with *r* as effect-size, without imputation (dangerousness, unpredictability, and responsibility).

However, there are methodological disadvantages to the imputation approach regarding comparability and estimation bias (Aloe, 2015; Roth, Le, Oh, van Iddekinge, & Bobko, 2018). To address these, the results were compared with average effect-sizes estimated separately from *r* (bivariate or partial) and *β* (Rosenthal & DiMatteo, 2001), if at least three studies were available. In the separate analyses (see online Supplementary Appendix 5), sensitivity analyses based on type of disorder and operationalisation were conducted. To assess publication bias, we conducted Funnel plots, Egger's regression, Trim and Fill procedure, and Fisher's Fail-Safe-N. Two subgroups of studies emerged based on common measures of continuum beliefs and analytic strategies [subgroup 1: one-item measure of continuum beliefs by Schomerus, Matschinger, and Angermeyer (2013) using regression analyses; subgroup 2: items of Thibodeau (2017, 2018, 2019) or Wiesjahn, Brabban, Jung, Gebauer, and Lincoln (2014), Wiesjahn, Jung, Kremser, Rief, and Lincoln, (2016) using correlation analyses], prompting us to do additional analyses referring to these subgroups.

## Results

**Fig. 1.**
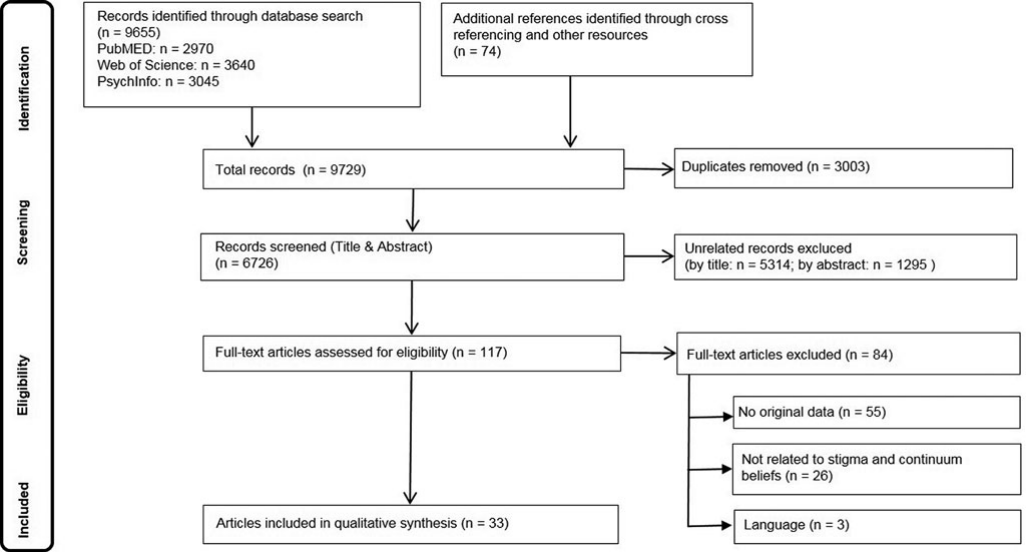
Flow chart of the review process.

Altogether, *N* = 6726 unique articles were identified after electronic database search, cross-referencing, and removal of duplicates. A total of 6609 records were excluded through title and abstract screening. Excluded studies focused on medical stigma (Al-Hazmi, 2015; Cacioppo & Gardner, 1993; Collins, 2006), topics only loosely related to mental health and illness, like well-being (Aggarwal & Sriram, 2018), classification issues (Casey et al., 2013) or the continuum within categories of specific mental disorders (Andrulonis, Glueck, Stroebel, & Vogel, 1982). A total of 117 full text articles were retrieved for further consideration, of which 84 records were discarded (Fig. 1). A considerable part of these were reviews, not excluded earlier to identify original studies from the respective reference lists. None of the reviews focused on continuum beliefs and stigma.

### Study characteristics

Descriptions of the included studies are provided in online Supplementary Appendix 3. Most studies were conducted in the United States (*n* = 10) and Germany (*n* = 12). Three studies were from Canada, two each were conducted in France and England, and one each in Singapore, the Netherlands, Ireland, and Japan. A total of 19 of the 33 studies originate from five research teams: Schomerus and Angermeyer (*n* = 6); Thibodeau (*n* = 4); Wiesjahn, Schlier, and Lincoln (*n* = 4); von dem Knesebeck and Makowski (*n* = 3); and Corrigan (*n* = 2). Twenty-five studies assessed attitudes of the general population and/or mental health professionals, experts, caregivers, etc. (*n* = 7) and/or people with mental illness (*n* = 4). Attitudes were assessed towards schizophrenia/psychosis (*n* = 19), depression (*n* = 11), alcohol use disorder (*n* = 3), obsessive-compulsive disorder (*n* = 2), posttraumatic stress disorder (*n* = 1), bipolar disorder (*n* = 1), and attention deficit hyperactivity disorder (*n* = 1) or mental illness in general (*n* = 6). Although there was no specific timeframe for eligibility, 27 of the included articles were published since 2015, thus attesting to the high actuality of the research field. The earliest included study was from 1957 (Cumming & Cumming, 1957), thus being the only included article published before 2003.

### Results of methodological quality assessment

The modified version of Downs & Black Quality Assessment (see online Supplementary Appendix 2) revealed an overall quality between 47% (Cumming & Cumming, 1957) and 100% (Speerforck et al., 2019) with 17 studies scoring 80% or higher.

### Assessment of continuum beliefs and stigma

Continuum beliefs were most frequently assessed with regards to case vignettes depicting a person with mental illness (18 out of 33 studies). Other methods included continuum beliefs elicited after other experimental manipulations (*n* = 4), surveys without using a vignette (*n* = 2), and qualitative studies (*n* = 3). Six intervention studies did not specifically measure continuum beliefs.

An easy to administer and simple measure to operationalise continuum beliefs (Schomerus et al., 2013) was used in eight studies: after having read a case vignette, respondents were asked to indicate their agreement with the following statement on Likert scales: ‘Basically we are all sometimes like [person X]. It is just a question how pronounced this state is’. Speerforck et al. (2019) added the aspect of ‘abnormality’: ‘All in all the problem of this person is abnormal’.

The 16-item Continuum Beliefs Questionnaire (CBQ; Wiesjahn et al., 2014) emphasises the normalcy of psychotic symptoms and was used in six studies, including a revised (Schlier, Scheunemann, & Lincoln, 2016) and an adapted version (Violeau, Valery, Fournier, & Prouteau, 2020). Thibodeau's assessment of continuum and categorical beliefs with varying numbers of items was used in five studies. Two studies used or adapted the Similar-Different-Scale (Corrigan, Bink, Fokuo, & Schmidt, 2015) and one study each used the Continuity with Normal Experience Scale (Norman, Windell, & Manchanda, 2012) and the Problem Drinking Belief Scale (Morris, Albery, Heather, & Moss, 2020).

Indicating a shared conceptualisation of stigma, the most frequently used measure of stigma is the Social Distance Scale (Link, Cullen, Frank, & Wozniak, 1987), assessing the desire to avoid people with certain characteristics in different social situations. Another commonly used scale is the Emotional Reactions to People with Mental Illness Scale (Angermeyer & Matschinger, 2003b; Angermeyer, Holzinger, & Matschinger, 2010), with subscales indicating pro-social reactions, fear, and anger. Measurement of stereotypes was more heterogeneous, with frequently [Stereotypes about Schizophrenia (Angermeyer & Matschinger, 2004); Semantic Differential Tool (Olmsted & Durham, 1976)] and rarely used inventories [Perceived Dangerousness Scale (Link et al., 1987), Explicit Measure of Self-Stereotype Association (Violeau et al., 2020)] and also heterogeneous subscales (dangerousness and/or unpredictability and responsibility/blame).

### Associations between continuum beliefs and stigma

Seven cross-sectional and six intervention studies investigate the associations between continuum beliefs and stigma-related outcomes. Relevant information was added by two qualitative studies, which mention the belief of similarity (‘they are folks just like us’) as a counter factor to stigma (Schoech, 2017). Interviewees explained a way to reduce social distance ‘is by understanding human experience as a continuum. This idea challenges the border between what's normal and abnormal by emphasizing that the difference is based on degrees, not absolute differences’ (Alvarado Chavarría, 2000, p. 94).

Several studies report a percentage of agreement to the continuum beliefs statement (‘Basically we are all sometimes like this person. It is just a question how pronounced this state is.’), collapsing the Likert-scale answers on each side of the midpoint into ‘agree’ and ‘disagree’ categories. For depression, 42–58% agreed with the continuum statement, while 14–25% disagreed; for schizophrenia only one in three persons agreed, while 40–50% disagreed (Angermeyer et al., 2015; Schomerus et al., 2013; Subramaniam et al., 2017). For ADHD, more than six times as many people agreed than disagreed (Speerforck et al., 2019); for alcohol use disorder, agreement rates were around one-third, while slightly more than 40% disagreed (Schomerus et al., 2013; Subramaniam et al., 2017). Hence, type of disorder proved relevant for the general population in perceiving disorders as relating with their own experiences to a varying degree. Concerning age and education, results are mixed with no clear tendency (Angermeyer et al., 2015; Schomerus et al., 2013; Speerforck et al., 2019; Subramaniam et al., 2017).

#### Overall effects

Table 1 shows an overview of meta-analytic findings for social distance (see also Table 2), emotional reactions (pro-social reactions, fear, and anger), and stereotypes (dangerousness, unpredictability, and responsibility). The combined correlation of continuum beliefs with stigmatising attitudes was mostly significant in the expected directions, with the exception of anger and responsibility, which were only significant for subgroups. Heterogeneity ranged from *I*^2^ = 0.00% to 91.86%. Analyses including only studies with similar operationalisation were less heterogeneous, indicating the influence of methodological differences. Heterogeneity was especially lower for ‘subgroup 2’, containing correlations and measures of Wiesjahn et al. and Thibodeau et al. Operationalisation also explained more heterogeneity than type of disorder. This pattern of results was even more apparent in the separate analyses of correlation and regression coefficients for social distance and pro-social reactions. Publication bias analyses did not indicate substantial bias. See online Supplementary Appendix 4 for meta-analyses of emotional reactions and stereotypes and online Supplementary Appendix 5 for separate meta-analyses.

**Table 1.** Overview of meta-analyses on the association of continuum beliefs and stigmatising attitudes (social distance, pro-social reactions, fear, anger, dangerousness, unpredictability, and responsibility)

Outcome	*k*	*r*	*p*	L CI	U CI	*I*^2^
Social distance	21	**−0.17**	<0.001	−0.22	−0.12	88.85
Depression	5	−0.11		−0.24	0.03	87.47
Schizophrenia	10	**−0.22**		−0.28	−0.14	86.26
Subgroup 1	13	**−0.14**		−0.21	−0.08	89.71
Subgroup 2	8	**−0.24**		−0.31	−0.17	61.78
Pro-social reactions	12	**0.15**	0.016	0.01	0.28	91.86
Depression	4	**0.21**		0.08	0.34	85.34
Schizophrenia	6	0.01		−0.32	0.35	95.30
Subgroup 1	8	**0.23**		0.15	0.30	91.12
Subgroup 2	4	−0.16		−0.61	0.36	88.58
Fear	13	**−0.07**	<0.001	−0.11	−0.03	76.82
Depression	4	−0.02		−0.12	0.08	75.81
Schizophrenia	7	**−0.12**		−0.17	−0.06	64.89
Subgroup 1	8	−0.05		−0.09	0.00	71.17
Subgroup 2	5	**−0.15**		−0.24	−0.06	29.63
Anger	12	−0.05	0.093	−0.01	0.10	77.01
Depression	4	0.01		−0.14	0.16	82.90
Schizophrenia	6	0.07		−0.05	0.18	75.52
Subgroup 1	8	**0.07**		0.01	0.13	79.63
Subgroup 2	4	−0.09		−0.20	0.02	00.00
Dangerousness^a^	8	**−0.12**	0.004	−0.21	−0.02	89.52
Schizophrenia^a^	7	**−0.14**		−0.23	−0.05	86.77
Subgroup 2^a^	6	**−0.21**		−0.33	−0.18	22.29
Unpredictability^a^	9	**−0.18**	<0.001	−0.28	−0.08	94.60
Schizophrenia^a^	7	**−0.19**		−0.29	−0.09	90.70
Subgroup 2^a^	6	**−0.26**		−0.33	−0.18	28.12
Responsibility^a^	4	−0.05	0.225	−0.18	0.08	85.72
Schizophrenia, subgroup 2^a^	3	**−0.10**		−0.11	−0.09	00.00

*Notes*. Outcome: overall results and subgroup analyses (type of disorder: depression, schizophrenia; methods: subgroup 1 = one-item measure of Schomerus et al., 2013, regression models; subgroup 2 = Thibodeau's/Wiesjahn's operationalisation, correlation models). *k*, number of effect-sizes; *r*, combined correlation coefficient, significant correlations highlighted in bold; *p*, two-tailed *p* value of combined *r*. L CI/U CI, lower and upper limit of confidence interval. *I*^2^, heterogeneity. Meta-analytic results of imputation method (Peterson & Brown, 2005).

a = no imputation method, *r* as effect-sizes.

**Table 2.** Meta-analysis and forest plot of the association of continuum beliefs and social distance (single study effect sizes and combined effect-size)

	Study	Disorder	*r*	L CI	U CI	Weight (%)	
1	Angermeyer et al. (2015)^1^	Depr.	−0.18	−0.25	−0.11	5.31	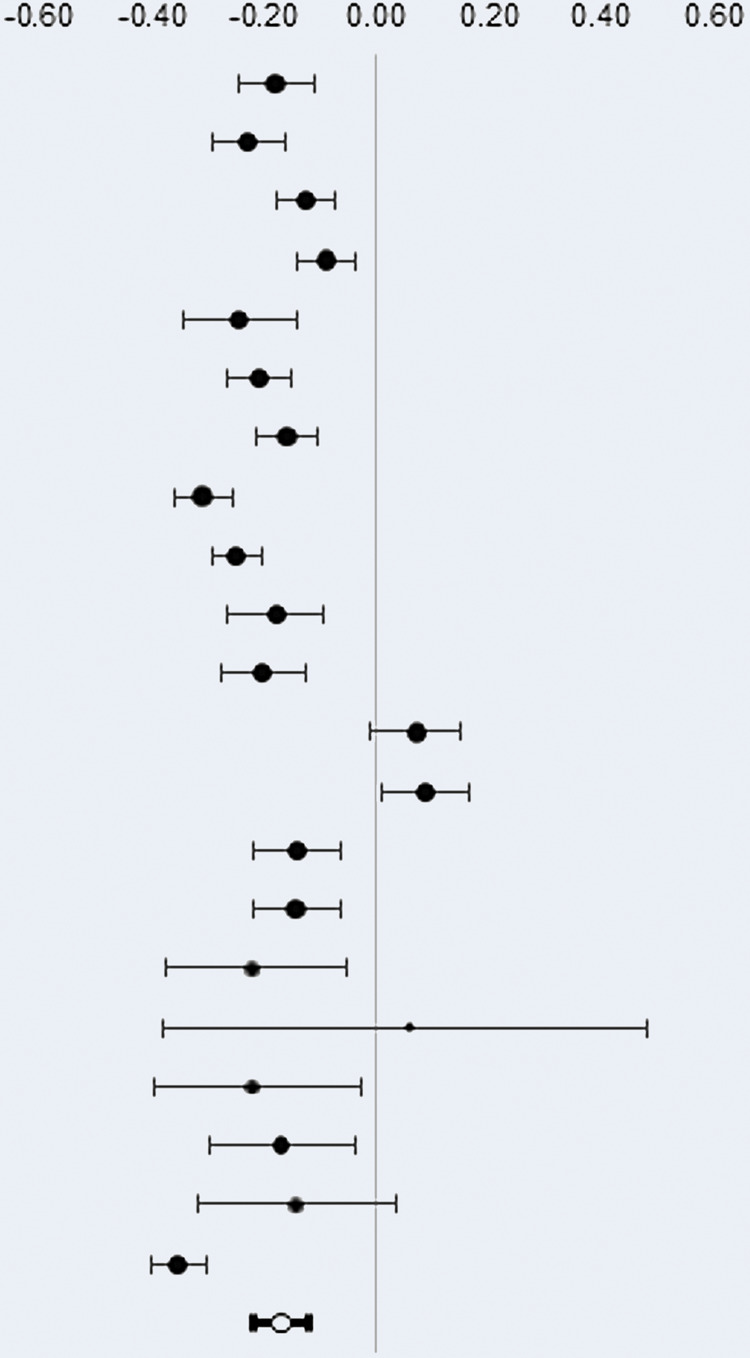
2	Angermeyer et al. (2015)^1^	Schiz.	−0.23	−0.29	−0.16	5.31	
3	Makowski et al. (2016a)^1^	Depr.	−0.13	−0.18	−0.07	5.53	
4	Makowski et al. (2016a)^1^	Schiz.	−0.09	−0.14	−0.04	5.54	
5	Schlier et al. (2016)^1^	Schiz.	−0.25	−0.34	−0.14	4.67	
6	Schomerus et al. (2013)^1^	Alc.	−0.21	−0.26	−0.15	5.48	
7	Schomerus et al. (2013)^1^	Depr.	−0.16	−0.22	−0.10	5.49	
8	Schomerus et al. (2013)^1^	Schiz.	−0.31	−0.36	−0.26	5.49	
9	Schomerus et al. (2016)^1^	Depr., Schiz.	−0.25	−0.29	−0.20	5.61	
10	Speerforck et al. (2019)^1^	ADHD	−0.18	−0.26	−0.09	5.00	
11	Subramaniam et al. (2017)^1^	Alc.	−0.20	−0.28	−0.12	5.15	
12	Subramaniam et al. (2017)^1^	Dement.	0.07	−0.01	0.15	5.14	
13	Subramaniam et al. (2017)^1^	Depr.	0.09	0.01	0.17	5.15	
14	Subramaniam et al. (2017)^1^	OCD	−0.14	−0.22	−0.06	5.15	
15	Subramaniam et al. (2017)^1^	Schiz.	−0.14	−0.22	−0.06	5.14	
16	Thibodeau and Peterson (2018)^2^	Schiz.	−0.22	−0.38	−0.05	3.54	
17	Thibodeau et al. (2018)^2^	Schiz.	0.06	−0.38	0.48	1.10	
18	Thibodeau (2017)^1^	Schiz.	−0.22	−0.40	−0.03	3.14	
19	Thibodeau (2019)^1^	Depr.	−0.17	−0.30	−0.04	4.20	
20	Wiesjahn et al. (2014)^1^	Schiz.	−0.15	−0.32	0.04	3.37	
21	Wiesjahn et al. (2016)^1^	Schiz.	−0.35	−0.40	−0.30	5.49	
			**−0.17**	**−0.22**	**−0.12**		

*Notes*: Population: 1 = general population, 2 = undergraduates; Disorder: Depr., depression; Schiz., schizophrenia; Alc., alcoholism; ADHD, attention deficit/hyperactivity disorder; Dement., dementia; OCD, obsessive-compulsive disorder; *r*, correlation coefficient; L CI/U CI, lower and upper limit of confidence interval. Weight, study weight. Forest plot: single study effect-sizes and combined effect-size with CI. Size of point reflects study weight.

#### Social distance

The overall correlation was significant: *r* = −0.17 (CI −0.22 to −0.12) indicating a small negative association. The subgroup analysis regarding type of disorder indicated a significant negative correlation particularly for schizophrenia (*r* = −0.22, CI −0.28 to −0.14). Most studies (Table 2) found consistent negative associations, except for an undergraduate (Thibodeau, Shanks, & Smith, 2018), dementia, and depression sample (Subramaniam et al., 2017).

The separate meta-analyses of *r* and *β* (*r*_p_) indicated significant effect-sizes from *r* = −0.19 to −0.24 and from *r*_p_ = −0.07 to −0.10. Heterogeneity was lower in the meta-analyses of *r* compared to *β* (*r*_p_). Subgroup analyses of Thibodeau's studies showed that applying a similar operationalisation yields homogeneous correlational estimates (*I*^2^ = 00.00). Partial correlational estimates, in contrast, were more homogeneous when different types of disorders were analysed separately.

#### Emotional reactions

Regarding the association with pro-social reactions, a significant positive correlation of *r* = 0.15 (CI 0.01–0.28) was found overall. Subgroup analyses regarding type of disorder were significant for depression, *r* = 0.21 (CI 0.08–0.34). Single effect-sizes vary from a majority of small positive correlations to three small and moderate negative correlations (Thibodeau, 2017; Thibodeau et al., 2018; Thibodeau & Peterson, 2018).

Two separate meta-analyses produced non-significant correlations. Sensitivity analyses showed more homogenous results for similar operationalisation. The partial correlation meta-analysis (*r*_p_ = 0.10) included different types of disorders, but with similar operationalisation and was homogenous.

Overall, fear was weakly negatively associated with continuum beliefs (Angermeyer et al., 2015; Makowski, Mnich, Angermeyer, & von dem Knesebeck, 2016a), yielding a significant combined correlation of *r* = −0.07 (CI −0.11 to −0.03). Subgroup analyses were significant for schizophrenia (*r* = −0.12, CI −0.17 to −0.06).

Regarding anger, results vary from significant small positive associations for depression and schizophrenia (Schomerus et al., 2013), a significant positive association for schizophrenia, but not for depression (Angermeyer et al., 2015), to non-significant associations (Makowski et al., 2016a). The combined effect size of *r* = −0.05 (CI −0.01 to 0.10) is insignificant as well as subgroup analyses regarding type of disorder.

#### Stereotypes

Dangerousness (*r* = −0.12, CI −0.21 to −0.02) and unpredictability (*r* = −0.18, CI −0.28 to −0.08) were overall small, but significantly and negatively correlated with continuum beliefs. These analyses are almost exclusively based on studies about schizophrenia. Heterogeneity was low for analyses with comparable methods in subgroup 2. The combined correlation for responsibility also yielded significance (*r* = −0.10, CI −0.11 to −0.09) and low heterogeneity, when only studies about schizophrenia were combined (Table 1).

### Intervention studies

Eight studies examined the effects of broader interventions, e.g. a mental health awareness campaign (Makowski et al., 2016b) or school-based project (Schulze, Richter-Werling, Matschinger, & Angermeyer, 2003) that explicitly used continuum messages, but also included others. Four of these studies focused on attitudes of professionals, one additional analysis of expert ratings did not specifically recommend continuum messages (Clement, Jarrett, Henderson, & Thornicroft, 2010). A programme for first responders found significant decreases in stigma combined across different sites (Dobson, Szeto, & Knaak, 2019; Szeto, Dobson, & Knaak, 2019). A psychoeducational therapy for caregivers (Shiraishi, Watanabe, Katsuki, Sakaguchi, & Akechi, 2019) and a workshop for mental health professionals found no significant effects on stigma (Helmus, Schaars, Wierenga, Glint, & van Os, 2019).

Studies investigating attitudes of general population samples could at least partially reduce stigma (Makowski et al., 2016b; Schulze et al., 2003), with the exception of Cumming and Cumming (1957). Since those studies did not, however, specifically manipulate continuum beliefs, any change in stigma that is attributable to altering continuum beliefs cannot exactly be determined.

Eleven studies investigated effects of specifically manipulating continuum beliefs and target attitudes of general population samples. In these studies, an unlabelled vignette fulfilling criteria of a mental illness is presented. The experimental manipulation typically consists of additional intervention texts (e.g. bogus scientific article and magazine article) or video, either presenting evidence for a continuum model or for distinct differences between mental health and illness, and a third neutral condition is used as a control. Table 3 contains an overview of stigma-related outcomes of continuum interventions compared to control conditions. All but one experimental manipulation did successfully change self-reported continuum beliefs in the intended way. Two studies found reduced desire for social distance in the continuum- compared to control conditions: *β* = 0.175, *p* = 0.003, reverse coded (Schomerus et al., 2016); *F*_(2,175)_ = 3.21, *p* = 0.02 (Cole & Warman, 2019). Furthermore, Thibodeau et al. (2018) found a marginal reduction compared to control, *F*_(1,66)_ = 3.14, *p* < 0.09, *η*_p_^2^ = 0.05, and a significant reduction compared to categorical condition. Four other studies did not find significant effects.

**Table 3.** Effects of intervention studies

Study	CB	SDS	Diff.	Unpred.	Danger	Blame	Pro-Social	Fear	Anger
Cole and Warman (2019)	▴	▾			▾	◊			
Corrigan et al. (2017)	▴		▾						
Dolphin and Hennessy (2017)	▴						◊	◊	▴
Morris et al. (2020)^a^	▴								
Schomerus et al. (2016)	▴	▾	▾	◊		▾			
Thibodeau (2017)	▴	◊	◊	◊	◊		◊	◊	◊
Thibodeau and Peterson (2018)	▴	◊	◊	◊	◊		◊	▴	◊
Thibodeau et al. (2018)	▴	◊	◊	◊	◊		◊	◊	◊
Thibodeau (2019)	▴	◊					◊	◊	◊
Violeau et al. (2020)	◊		▾	▴	◊				
Wiesjahn et al. (2016)	▴	◊		◊	◊	◊		◊	

Continuum belief intervention in comparison with the control group.

*Notes*: CB, continuum Belief; SDS, Social Distance Scale; Diff., difference measures; Unpred., unpredictability; Pro-Social, pro-social reactions; ▴, significant increase; ▾, significant decrease; ◊, no significant findings.

a No direct stigma measures.

Perceptions of difference were lower in the continuum- compared to neutral or categorical conditions in three studies. For negative stereotypes, results are inconsistent. There were significant effects on unpredictability: CB: *M* = 5.5, Control: *M* = 4.43, *t*_(359)_ = 3.24, *p* = 0.004 (Violeau et al., 2020), as well as compared to categorical (Thibodeau et al., 2018) and biogenetic conditions (Wiesjahn, Jung, Kremser, Rief, & Lincoln, 2016). For dangerousness, effects have been found pre to post, *t*_(58)_ = 3.10, *p* = 0.003 (Cole & Warman, 2019), as well as compared to the categorical condition (Thibodeau et al., 2018).

For blame Schomerus et al. (2016) found a decrease compared to control group (*β* = −0.178, *p* = 0.05), while other studies found no effects or even an increase in blame compared to the categorical condition (Wiesjahn et al., 2016).

No positive effects were found for emotional reactions (pro-social, fear, and anger). On the contrary, there was a significant main effect for condition for within category assimilation anger (*F*_(2,150)_ = 5.44, *p* = 0.005; Dolphin and Hennessy, 2017) and an increase in fear compared to the control group (*F*_(1,118)_ = 5.73, *p* = 0.018, *η*_p_^2^ = 0.05; Thibodeau and Peterson, 2018). Compared to the categorical group, less pro-social reactions were found (Thibodeau et al., 2018), but most studies reported no significant changes (Table 3).

Regarding studies about persons with mental illness, Thibodeau (2019) investigated attitudes of people with self-reported depression and found no significant intervention effects on depression stigma. Beyond stigmatising attitudes, Morris et al. (2020) found a continuum intervention on alcohol use disorder to improve problem recognition of the respondents' own heavy drinking.

To sum up, there is evidence of successful manipulations of continuum beliefs, but mixed evidence concerning changes in stigma through manipulation of continuum beliefs with no apparent tendency regarding the type of investigated mental illness. Evidence is scant for groups other than the general population. We will discuss how methodological differences between studies might have contributed to these seemingly contradictory outcomes.

## Discussion

This is the first systematic review and meta-analysis to investigate the associations of continuum beliefs of mental health and illness with mental health stigma, demonstrating that continuum beliefs are generally associated with lower desire for social distance, lower perception of dangerousness and unpredictability as well as less fear and more pro-social reactions.

Until now, continuum beliefs have most frequently been assessed with regards to depression and schizophrenia, with higher general agreement to continuum beliefs for depression. Associations between continuum beliefs and stigma are similar for all investigated types of disorders, but also show illness-related differences. For schizophrenia, findings are most consistent throughout all conducted meta-analyses. For depression, continuum beliefs are significantly associated with more pro-social reactions. The lack of associations with social distance or fear in depression could represent a ceiling effect. Associations are most robust where stigma is most severe (Angermeyer & Matschinger, 2003a, 2003b). Future studies might add on by adapting stigma measures to the investigated mental illness, facets of stigma play different roles in different disorders.

Regarding intervention studies, experimental designs are successfully manipulating continuum beliefs, but this is inconsistently accompanied by changes in stigma. Since few studies showed insignificant or even opposite findings, with some continuum interventions increasing stigma, the question arises which methodological differences are responsible for these divergent findings.

One general difference appears to be whether respondents are encouraged to see *themselves* on a continuum, or if they are asked (or instructed) about a continuum in more general terms, without reference to themselves. This seems to echo through operationalisation as well as design of interventions. To what extent respondents perceive people with mental illness as in-group or out-group (Tajfel & Turner, 1979) while they complete questionnaires and interventions might influence self-reported stigma.

Continuum beliefs can be elicited by establishing a personal connection between respondents and vignette character. This is exemplified in the item ‘Basically we are all sometimes like this person. It is just a question how pronounced this state is’ (Schomerus et al., 2013). By suggesting similarity between ‘us’ and ‘them’, it measures the respondent's willingness to accept the depicted person as one of ‘us’. Studies using this type of operationalisation generally show results in line with the hypotheses. Alternatively, continuum beliefs can be framed in more general terms, as a continuum between a state of illness and normalcy, not directly involving the respondents. Along this line, Thibodeau's set of items contain more general descriptions not including the respondent (e.g. ‘People who have schizophrenia have symptoms [delusions, hallucinations] that are similar to the occasional experiences of ordinary people’). Therefore, these items can be answered affirmatively, even if people with mental illness are regarded as an out-group, probably leading to more heterogeneous results. Our review suggests that personally relating respondents to a continuum, rather than informing them on the concept, could be crucial to using continuum messages for de-stigmatisation.

Manipulations of intervention studies are prominently text-based, which is of high internal validity and can easily be implemented into online surveys. Undergoing efforts to further improve continuum interventions, by designing audio-visual vignettes (Corrigan et al., 2015, 2017; Dolphin & Hennessy, 2017; Morris et al., 2020), or extending the intervention to more time points (Corrigan et al., 2015; Thibodeau, 2019; Thibodeau et al., 2018) are likely to improve personal involvement of the respondent as well. Personal contact and the inclusion of lived experiences had positive effects (Hansson, Stjernswärd, & Svensson, 2016). Corrigan et al. (2017) combined these factors into a video intervention connoting personal contact to a person with schizophrenia. Other factors to improve identification could involve the gender of the vignette character (Angermeyer et al., 2015), as well as cultural, ethnic, and national characteristics (Corrigan, 2004). Certainly, face-to-face contact would improve the respondent's personal involvement (Thibodeau, 2019), however, our results indicate that text- or video-based interventions are also capable of promoting a continuum of mental health and illness which includes the respondents and encourages them to view people with mental illness as in-group. In line with that, studies should focus on further developing even more identifiable and involving interventions. Various promising e-mental-health approaches have been detected to increase engagement and improve identification with fictional characters (Feltz, Forlenza, Winn, & Kerr, 2014; Igartua & Frutos, 2017). The most seminal approach to improve engagement is gamification, where game elements such as avatars, storylines, and rewarding feedback are implemented into non-game contexts (Looyestyn et al., 2017; Miller & Polson, 2019; Sardi, Idri, & Fernández-Alemán, 2017; Schwarz, Huertas-Delgado, Cardon, & DeSmet, 2020). Current virtualisation and digitalisation should be appreciated to create personally involving, virtual contact interventions and foster perceptions of people with mental illness as being in-group and by that improve attitudes towards mental illness.

### Methodological considerations

Our review shows that continuum beliefs and stigma have mostly been examined for only two disorders, depression and schizophrenia, while investigation of other disorders such as SUD is only beginning. Also, most studies are from two countries only, so any conclusions are limited to the disorders and countries studied.

In this emerging field, we aimed to arrive at a comprehensive review of literature, also including interventions not primarily focusing on continuum beliefs. However, it is likely that more interventions might have promulgated a continuum model of mental health and illness, but without explicitly mentioning it or including appropriate measures, they could not be included in the review. We did not establish formal reliability of our screening of articles but aimed at being over-inclusive in the screening stage and resolving all conflicts regarding eligibility among four of the authors based on the full texts.

Low initial stigma prior to the interventions could have caused a ceiling effect and needs to be considered as a reason for low effect-sizes (Schulze et al., 2003; Shiraishi et al., 2019). Most available studies obtained subjective information through self-ratings. Participants rarely state negative attitudes, especially if stigma reduction is the obvious study aim (Helmus et al., 2019). Almost all included articles discuss social desirability and resulting limited validity. Nevertheless, studies that record attitudes through implicit assessment (Schlier & Lincoln, 2019) or measurable behaviour, e.g. seating distance (Thibodeau, 2017), are exceptions.

The included studies show high heterogeneity, contain real variance (Borenstein, 2009) and need to be considered inconsistent. To account for high *I*^2^, three explanations are suggested: (a) methodological subgroups, (b) choice of effect measures, and (c) clinically important subgroups (Higgins et al., 2003). (a) In our analyses, methodological differences (workgroups, questionnaires, etc.) explain heterogeneity best. (b) Regarding statistical methods, imputing *β* into *r*-meta-analysis might have introduced variance, because *β* usually contains influences of covariates. The use of two different meta-analytic approaches enables us to understand our data the best: separate meta-analyses of studies reporting *r* or *β* yielded valuable information (Aloe, 2015). Lower heterogeneity in separate meta-analyses compared to using (imputed) *r* lead to the finding that heterogeneity is not completely inherent to the study contents but partly due to statistical methods. (c) Subgroup and sensitivity analyses regarding the type of disorder explained rather little heterogeneity. Interpretation needs to be done with caution due to the low number of studies (Thompson & Higgins, 2002). We established a measure of methodological quality but did not add it as a covariate to our analyses. Methodological quality is generally sensitive to bias in the selection of criteria and coding, and mostly limited to the published content of the paper (Higgins & Green, 2011). Also, moderator analyses are sensitive to selection of articles (Van Rhee, Suurmond, & Hak, 2015), so we considered methodological quality at present more suitable for narrative rather than statistical analyses.

Based on findings so far, future investigations should focus on the following: first, a theoretically based investigation of further influencing variables such as previous contact, age, or gender seems necessary. Second, continuum beliefs should be tested in relation to the construct of social identity (Tajfel & Turner, 1979; Turner & Oakes, 1986), to investigate perceptions of in-/out-group together with continuum belief interventions. Finally, to further improve interventional outcomes, the effects of different interventions should be assessed with special focus on how respondents are personally involved with the concept of a continuum. Digital- and gamification interventions, long-term interventions and the comparison regarding types of mental disorders should be considered to create even more effective interventions reducing stigma and improving help-seeking.

In summary, continuum beliefs can be acknowledged as beneficial to be included in anti-stigma campaigns and interventions with promising effects on subsequent health-related outcomes. Certainly, continuum beliefs alone will not be able to solve the problem of mental health-related stigma, yet might be able to provide an inclusive and promising foundation for other intervention messages. Therefore, we regard it a contemporary and necessary conclusion to include the concept of continuum beliefs into future study on destigmatising mental illness.

## Supplementary material

For supplementary material accompanying this paper visit https://doi.org/10.1017/S0033291721000854. click here to view supplementary material

## References

[ref1] Aggarwal, P., & Sriram, S. (2018). Exploring well-being among mental health professionals in India. Psychological Studies, 63(4), 335–345. doi: 10.1007/s12646-018-0470-x.

[ref2] Al-Hazmi, A. H. (2015). Knowledge, attitudes and practice of dentists concerning the occupational risks of hepatitis B virus in Al Jouf Province, Saudi Arabia. Nigerian Journal of Clinical Practice, 18(2), 276–281. doi: 10.4103/1119-3077.151067. 25666007

[ref3] Aloe, A. M. (2015). Inaccuracy of regression results in replacing bivariate correlations. Research Synthesis Methods, 6(1), 21–27. doi: 10.1002/jrsm.1126. 26035467

[ref4] Alvarado Chavarría, M. (2000). Let's try to change it: Psychiatric stigmatization, Consumer/Survivor activism, and the Link and Phelan model. doi: 10.15760/etd.904.

[ref5] Andrulonis, P. A., Glueck, B. C., Stroebel, C. F., & Vogel, N. G. (1982). Borderline personality subcategories. The Journal of Nervous and Mental Disease, 170(11), 670–679. doi: 10.1097/00005053-198211000-00006. 7119767

[ref6] Angermeyer, M. C., Holzinger, A., & Matschinger, H. (2010). Emotional reactions to people with mental illness. Epidemiologia e Psichiatria Sociale, 19(1), 26–32. doi: 10.1017/S1121189X00001573. 20486421

[ref7] Angermeyer, M. C., & Matschinger, H. (2003a). Public beliefs about schizophrenia and depression: Similarities and differences. Social Psychiatry and Psychiatric Epidemiology, 38(9), 526–534. doi: 10.1007/s00127-003-0676-6. 14504738

[ref8] Angermeyer, M. C., & Matschinger, H. (2003b). The stigma of mental illness: Effects of labelling on public attitudes towards people with mental disorder. Acta Psychiatrica Scandinavica, 108(4), 304–309. doi: 10.1034/j.1600-0447.2003.00150.x. 12956832

[ref9] Angermeyer, M. C., & Matschinger, H. (2004). The stereotype of schizophrenia and its impact on discrimination against people with schizophrenia: Results from a representative survey in Germany. Schizophrenia Bulletin, 30(4), 1049–1061. doi: 10.1093/oxfordjournals.schbul.a007120. 15954207

[ref10] Angermeyer, M. C., Millier, A., Rémuzat, C., Refaï, T., Schomerus, G., & Toumi, M. (2015). Continuum beliefs and attitudes towards people with mental illness: Results from a national survey in France. The International Journal of Social Psychiatry, 61(3), 297–303. doi: 10.1177/0020764014543312. 25061023

[ref11] Angermeyer, M. C., van der Auwera, S., Carta, M. G., & Schomerus, G. (2017). Public attitudes towards psychiatry and psychiatric treatment at the beginning of the 21st century: A systematic review and meta-analysis of population surveys. World Psychiatry, 16(1), 50–61. doi: 10.1002/wps.20383. 28127931 PMC5269489

[ref12] Borenstein, M. (2009). Introduction to meta-analysis. Chichester, UK: John Wiley & Sons.

[ref13] Cacioppo, J. T., & Gardner, W. L. (1993). What underlies medical donor attitudes and behavior? Health Psychology, 12(4), 269–271. doi: 10.1037/0278-6133.12.4.269. 8404799

[ref14] Casey, B. J., Craddock, N., Cuthbert, B. N., Hyman, S. E., Lee, F. S., & Ressler, K. J. (2013). DSM-5 and RDoC: Progress in psychiatry research? Nature Reviews. Neuroscience, 14(11), 810–814. doi: 10.1038/nrn3621. 24135697 PMC4372467

[ref15] Clement, S., Jarrett, M., Henderson, C., & Thornicroft, G. (2010). Messages to use in population-level campaigns to reduce mental health-related stigma: Consensus development study. Epidemiology and Psychiatric Sciences, 19(1), 72–79. doi: 10.1017/s1121189x00001627. 20486426

[ref16] Clement, S., Schauman, O., Graham, T., Maggioni, F., Evans-Lacko, S., Bezborodovs, N., & Thornicroft, G. (2015). What is the impact of mental health-related stigma on help-seeking? A systematic review of quantitative and qualitative studies. Psychological Medicine, 45(1), 11–27. doi: 10.1017/S0033291714000129. 24569086

[ref17] Cole, J. L., & Warman, D. M. (2019). An examination of Continuum Beliefs Versus Biogenetic beliefs in reducing stigma toward violent intrusive thoughts in OCD. Journal of Obsessive-Compulsive and Related Disorders, 23, 100478. doi: 10.1016/j.jocrd.2019.100478.

[ref18] Collins, P. Y. (2006). Challenges to HIV prevention in psychiatric settings: Perceptions of South African mental health care providers. Social Science & Medicine (1982), 63(4), 979–990. doi: 10.1016/j.socscimed.2006.03.003 16647793 PMC2800079

[ref19] Corrigan, P. W. (2004). Don't call me nuts: An international perspective on the stigma of mental illness. Acta Psychiatrica Scandinavica, 109(6), 403–404. doi: 10.1111/j.1600-0047.2004.00316.x. 15117284

[ref20] Corrigan, P. W., Bink, A. B., Fokuo, J. K., & Schmidt, A. (2015). The public stigma of mental illness means a difference between you and me. Psychiatry Research, 226(1), 186–191. doi: 10.1016/j.psychres.2014.12.047. 25660735

[ref21] Corrigan, P. W., Schmidt, A., Bink, A. B., Nieweglowski, K., Al-Khouja, M. A., Qin, S., & Discont, S. (2017). Changing public stigma with continuum beliefs. Journal of Mental Health (Abingdon, England), 26(5), 411–418. doi: 10.1080/09638237.2016.1207224. 27461413

[ref22] Cumming, J., & Cumming, E. (1957). Closed ranks: An experiment in mental health education. Commonwealth fund publications. Cambridge: Harvard University Press. doi: 10.4159/harvard.9780674491779.

[ref23] Dobson, K. S., Szeto, A., & Knaak, S. (2019). The working mind: A meta-analysis of a workplace mental health and stigma reduction program. Canadian Journal of Psychiatry. Revue Canadienne De Psychiatrie, 64(Suppl. 1), 39S–47S. doi: 10.1177/0706743719842559. 31122049 PMC6591747

[ref24] Dolphin, L., & Hennessy, E. (2017). Labelling effects and adolescent responses to peers with depression: An experimental investigation. BMC Psychiatry, 17(1), 228. doi: 10.1186/s12888-017-1389-9. 28646873 PMC5483288

[ref25] Downs, S. H., & Black, N. (1998). The feasibility of creating a checklist for the assessment of the methodological quality both of randomised and non-randomised studies of health care interventions. Journal of Epidemiology and Community Health, 52(6), 377–384. doi: 10.1136/jech.52.6.377. 9764259 PMC1756728

[ref26] Eisenberg, D., Downs, M. F., Golberstein, E., & Zivin, K. (2009). Stigma and help seeking for mental health among college students. Medical Care Research and Review: MCRR, 66(5), 522–541. doi: 10.1177/1077558709335173. 19454625

[ref27] Feltz, D. L., Forlenza, S. T., Winn, B., & Kerr, N. L. (2014). Cyber buddy is better than no buddy: A test of the Köhler motivation effect in exergames. Games for Health Journal, 3(2), 98–105. doi: 10.1089/g4h.2013.0088. 26196051

[ref28] Freeman, M. (2005). WHO resource book on mental health, human rights and legislation. World Health Organization. Retrieved from http://gbv.eblib.com/patron/FullRecord.aspx?p=284650.

[ref29] Goldberg, D. (2000). Plato versus Aristotle: Categorical and dimensional models for common mental disorders. Comprehensive Psychiatry, 41(2), 8–13. doi: 10.1016/S0010-440X(00)80002-4. 10746898

[ref30] Hansson, L., Stjernswärd, S., & Svensson, B. (2016). Changes in attitudes, intended behaviour, and mental health literacy in the Swedish population 2009–2014: An evaluation of a national antistigma programme. Acta Psychiatrica Scandinavica, 134(Suppl. 446), 71–79. doi: 10.1111/acps.12609. 27426648

[ref31] Helmus, K., Schaars, I. K., Wierenga, H., de Glint, E., & van Os, J. (2019). Decreasing stigmatization: Reducing the discrepancy between ‘Us’ and ‘them’. An intervention for mental health care professionals. Frontiers in Psychiatry, 10, 243. doi: 10.3389/fpsyt.2019.00243. 31214053 PMC6555228

[ref32] Henderson, C., Evans-Lacko, S., & Thornicroft, G. (2013). Mental illness stigma, help seeking, and public health programs. American Journal of Public Health, 103(5), 777–780. doi: 10.2105/AJPH.2012.301056. 23488489 PMC3698814

[ref33] Higgins, J. P. T., & Green, S. (eds) (2011) Cochrane handbook for systematic reviews of interventions Version 5.1.0 (updated March 2011). The Cochrane Collaboration. Retrieved from http://www.handbook.cochrane.org.

[ref34] Higgins, J. P. T., Thompson, S. G., Deeks, J. J., & Altman, D. G. (2003). Measuring inconsistency in meta-analyses. BMJ (Clinical Research Ed.), 327(7414), 557–560. doi: 10.1136/bmj.327.7414.557. PMC192859 12958120

[ref35] Huedo-Medina, T. B., Sánchez-Meca, J., Marín-Martínez, F., & Botella, J. (2006). Assessing heterogeneity in meta-analysis: Q statistic or I^2^ index? Psychological Methods, 11(2), 193–206. doi: 10.1037/1082-989X.11.2.193. 16784338

[ref36] Igartua, J. J., & Frutos, F. J. (2017). Enhancing attitudes toward stigmatized groups with movies: Mediating and moderating processes of narrative persuasion. International Journal of Communication, 11(20), 158–177.

[ref37] Lasalvia, A., Zoppei, S., van Bortel, T., Bonetto, C., Cristofalo, D., Wahlbeck, K., & Thornicroft, G. (2013). Global pattern of experienced and anticipated discrimination reported by people with major depressive disorder: A cross-sectional survey. The Lancet, 381(9860), 55–62. doi: 10.1016/S0140-6736(12)61379-8. 23083627

[ref38] Link, B. G., Cullen, F. T., Frank, J., & Wozniak, J. F. (1987). The social rejection of former mental patients: Understanding why labels matter. American Journal of Sociology, 92(6), 1461–1500. doi: 10.1086/228672.

[ref39] Link, B. G., & Phelan, J. C. (2001). Conceptualizing stigma. Annual Review of Sociology, 27(1), 363–385. doi: 10.1146/annurev.soc.27.1.363.

[ref40] Looyestyn, J., Kernot, J., Boshoff, K., Ryan, J., Edney, S., & Maher, C. (2017). Does gamification increase engagement with online programs? A systematic review. PLoS One, 12(3), e0173403. doi: 10.1371/journal.pone.0173403. 28362821 PMC5376078

[ref41] Makowski, A. C., Mnich, E. E., Angermeyer, M. C., & von dem Knesebeck, O. (2016a). Continuum beliefs in the stigma process regarding persons with schizophrenia and depression: Results of path analyses. PeerJ, 4, e2360. doi: 10.7717/peerj.2360. 27703840 PMC5045891

[ref42] Makowski, A. C., Mnich, E. E., Ludwig, J., Daubmann, A., Bock, T., Lambert, M., … von dem Knesebeck, O. (2016b). Changes in beliefs and attitudes toward people with depression and schizophrenia – results of a public campaign in Germany. Psychiatry Research, 237, 271–278. doi: 10.1016/j.psychres.2016.01.029. 26805560

[ref43] McGowan, J., Sampson, M., Salzwedel, D. M., Cogo, E., Foerster, V., & Lefebvre, C. (2016). PRESS peer review of electronic search strategies: 2015 guideline statement. Journal of Clinical Epidemiology, 75, 40–46. doi: 10.1016/j.jclinepi.2016.01.021. 27005575

[ref44] Miller, E., & Polson, D. (2019). Apps, avatars, and robots: The future of mental healthcare. Issues in Mental Health Nursing, 40(3), 208–214. doi: 10.1080/01612840.2018.1524535. 30605359

[ref45] Moher, D., Liberati, A., Tetzlaff, J., & Altman, D. G. (2009). Preferred reporting items for systematic reviews and meta-analyses: The PRISMA statement. Journal of Clinical Epidemiology, 62(10), 1006–1012. doi: 10.1016/j.jclinepi.2009.06.005. 19631508

[ref46] Morris, J., Albery, I. P., Heather, N., & Moss, A. C. (2020). Continuum beliefs are associated with higher problem recognition than binary beliefs among harmful drinkers without addiction experience. Addictive Behaviors, 105, 106292. doi: 10.1016/j.addbeh.2020.106292. 32007833

[ref47] Norman, R. M. G., Windell, D., & Manchanda, R. (2012). Examining differences in the stigma of depression and schizophrenia. The International Journal of Social Psychiatry, 58(1), 69–78. doi: 10.1177/0020764010387062. 21088035

[ref48] Olmsted, D. W., & Durham, K. (1976). Stability of mental health attitudes: A semantic differential study. Journal of Health and Social Behavior, 17(1), 35–44. 1270784

[ref49] Peterson, R. A., & Brown, S. P. (2005). On the use of beta coefficients in meta-analysis. The Journal of Applied Psychology, 90(1), 175–181. doi: 10.1037/0021-9010.90.1.175. 15641898

[ref50] wRiley, R. D., Higgins, J. P. T., & Deeks, J. J. (2011). Interpretation of random effects meta-analyses. BMJ (Clinical Research Ed), 342, d549. doi: 10.1136/bmj.d549. 21310794

[ref51] Rosenthal, R., & DiMatteo, M. R. (2001). Meta-analysis: Recent developments in quantitative methods for literature reviews. Annual Review of Psychology, 52, 59–82. doi: 10.1146/annurev.psych.52.1.59. 11148299

[ref52] Roth, P. L., Le, H., Oh, I‑S, van Iddekinge, C. H., & Bobko, P. (2018). Using beta coefficients to impute missing correlations in meta-analysis research: Reasons for caution. The Journal of Applied Psychology, 103(6), 644–658. doi: 10.1037/apl0000293. 29369653

[ref53] Sardi, L., Idri, A., & Fernández-Alemán, J. L. (2017). A systematic review of gamification in e-Health. Journal of Biomedical Informatics, 71, 31–48. doi: 10.1016/j.jbi.2017.05.011. 28536062

[ref54] Schewe, A. F., Hülsheger, U. R., & Maier, G. W. (2014). Metaanalyse–praktische Schritte und Entscheidungen im Umsetzungsprozess. Zeitschrift Für Arbeits- Und Organisationspsychologie A&O, 58(4), 186–205. doi: 10.1026/0932-4089/a000165.

[ref55] Schlier, B., & Lincoln, T. M. (2019). The stigma of mental illness: Testing for the implicit bias in diagnostic labels. Psychiatry Research, 275, 221–227. doi: 10.1016/j.psychres.2019.03.028. 30928725

[ref56] Schlier, B., Scheunemann, J., & Lincoln, T. M. (2016). Continuum beliefs about psychotic symptoms are a valid, unidimensional construct: Construction and validation of a revised continuum beliefs questionnaire. Psychiatry Research, 241, 147–153. doi: 10.1016/j.psychres.2016.04.085. 27175910

[ref57] Schoech (2017). Clinicians’ Perceptions of Trauma Treatment for People with Intellectual Disabilities. Walden Dissertations and Doctoral Studies. Retrieved from https://scholarworks.waldenu.edu/dissertations/4171/.

[ref58] Schomerus, G., Angermeyer, M. C., Baumeister, S. E., Stolzenburg, S., Link, B. G., & Phelan, J. C. (2016). An online intervention using information on the mental health-mental illness continuum to reduce stigma. European Psychiatry: The Journal of the Association of European Psychiatrists, 32, 21–27. doi: 10.1016/j.eurpsy.2015.11.006. 26802980

[ref59] Schomerus, G., Matschinger, H., & Angermeyer, M. C. (2013). Continuum beliefs and stigmatizing attitudes towards persons with schizophrenia, depression and alcohol dependence. Psychiatry Research, 209(3), 665–669. doi: 10.1016/j.psychres.2013.02.006. 23465293

[ref60] Schulze, L. N., Klinger-König, J., Stolzenburg, S., Wiese, J., Speerforck, S., van der Auwera-Palitschka, S., & Schomerus, G. (2020). Shame, self-identification with having a mental illness, and willingness to seek help in northeast Germany. Psychiatry Research, 285, 112819. doi: 10.1016/j.psychres.2020.112819. 32036156

[ref61] Schulze, B., Richter-Werling, M., Matschinger, H., & Angermeyer, M. C. (2003). Crazy? So what! Effects of a school project on students’ attitudes towards people with schizophrenia. Acta Psychiatrica Scandinavica, 107(2), 142–150. doi: 10.1034/j.1600-0447.2003.02444.x. 12534440

[ref62] Schwarz, A. F., Huertas-Delgado, F. J., Cardon, G., & DeSmet, A. (2020). Design features associated with user engagement in digital games for healthy lifestyle promotion in youth: A systematic review of qualitative and quantitative studies. Games for Health Journal, 9(3), 150–163. doi: 10.1089/g4h.2019.0058. 31923363

[ref63] Shiraishi, N., Watanabe, N., Katsuki, F., Sakaguchi, H., & Akechi, T. (2019). Effectiveness of the Japanese standard family psychoeducation on the mental health of caregivers of young adults with schizophrenia: A randomised controlled trial. BMC Psychiatry, 19(1), 263. doi: 10.1186/s12888-019-2252-y. 31477061 PMC6719377

[ref64] Speerforck, S., Stolzenburg, S., Hertel, J., Grabe, H. J., Strauß, M., Carta, M. G., & Schomerus, G. (2019). ADHD, stigma and continuum beliefs: A population survey on public attitudes towards children and adults with attention deficit hyperactivity disorder. Psychiatry Research, 282, 112570. doi: 10.1016/j.psychres.2019.112570. 31558401

[ref65] Subramaniam, M., Abdin, E., Picco, L., Shahwan, S., Jeyagurunathan, A., Vaingankar, J. A., & Chong, S. A. (2017). Continuum beliefs and stigmatising beliefs about mental illness: Results from an Asian community survey. BMJ Open, 7(4), e014993. doi: 10.1136/bmjopen-2016-014993. PMC5594210 28381420

[ref66] Suurmond, R., van Rhee, H., & Hak, T. (2017). Introduction, comparison, and validation of meta-essentials: A free and simple tool for meta-analysis. Research Synthesis Methods, 8(4), 537–553. doi: 10.1002/jrsm.1260. 28801932 PMC5725669

[ref67] Szeto, A., Dobson, K. S., & Knaak, S. (2019). The road to mental readiness for first responders: A meta-analysis of program outcomes. Canadian Journal of Psychiatry. Revue Canadienne De Psychiatrie, 64(Suppl. 1), 18S–29S. doi: 10.1177/0706743719842562. 31010293 PMC6591743

[ref68] Tajfel, H., & Turner, J. C. (1979). An integrative theory of intergroup conflict. In W. G. Austin & S. Worchel (Eds.), The social psychology of intergroup relations (pp. 33–47). Monterey: Brooks-Cole.

[ref69] Thibodeau, R. (2017). Continuum beliefs and schizophrenia stigma: Correlational and experimental evidence. Stigma and Health, 2(4), 266–270. doi: 10.1037/sah0000061.

[ref70] Thibodeau, R. (2019). Continuum belief, categorical belief, and depression stigma: Correlational evidence and outcomes of an online intervention. Stigma and Health. Advance online publication. doi: 10.1037/sah0000211.

[ref71] Thibodeau, R., & Peterson, K. M. (2018). On continuum beliefs and psychiatric stigma: Similarity to a person with schizophrenia can feel too close for comfort. Psychiatry Research, 270, 731–737. doi: 10.1016/j.psychres.2018.10.070. 30551317

[ref72] Thibodeau, R., Shanks, L. N., & Smith, B. P. (2018). Do continuum beliefs reduce schizophrenia stigma? Effects of a laboratory intervention on behavioral and self-reported stigma. Journal of Behavior Therapy and Experimental Psychiatry, 58, 29–35. doi: 10.1016/j.jbtep.2017.08.002. 28803131

[ref73] Thompson, S. G., & Higgins, J. P. T. (2002). How should meta-regression analyses be undertaken and interpreted? Statistics in Medicine, 21(11), 1559–1573. doi: 10.1002/sim.1187. 12111920

[ref74] Thornicroft, G., Brohan, E., Rose, D., Sartorius, N., & Leese, M. (2009). Global pattern of experienced and anticipated discrimination against people with schizophrenia: A cross-sectional survey. The Lancet, 373(9661), 408–415. doi: 10.1016/S0140-6736(08)61817-6. 19162314

[ref75] Turner, J. C., & Oakes, P. J. (1986). The significance of the social identity concept for social psychology with reference to individualism, interactionism and social influence. British Journal of Social Psychology, 25(3), 237–252. doi: 10.1111/j.2044-8309.1986.tb00732.x.

[ref76] Van Rhee, H. J., Suurmond, R., & Hak, T. (2015). User manual for Meta-Essentials: Workbooks for meta-analysis (Version 1.4) Rotterdam, The Netherlands: Erasmus Research Institute of Management. Retrieved from http://www.erim.eur.nl/research-support/meta-essentials.

[ref77] Violeau, L., Valery, K.‑M., Fournier, T., & Prouteau, A. (2020). How continuum beliefs can reduce stigma of schizophrenia: The role of perceived similarities. Schizophrenia Research, 220, 46–53. 10.1016/j.schres.2020.04.014. 32354661

[ref78] Wiesjahn, M., Brabban, A., Jung, E., Gebauer, U. B., & Lincoln, T. M. (2014). Are continuum beliefs about psychotic symptoms associated with stereotypes about schizophrenia? Psychosis, 6(1), 50–60. doi: 10.1080/17522439.2012.740068.

[ref79] Wiesjahn, M., Jung, E., Kremser, J. D., Rief, W., & Lincoln, T. M. (2016). The potential of continuum versus biogenetic beliefs in reducing stigmatization against persons with schizophrenia: An experimental study. Journal of Behavior Therapy and Experimental Psychiatry, 50, 231–237. doi: 10.1016/j.jbtep.2015.09.007. 26412294

