# Supplementary material for: Continuum beliefs and mental illness stigma: a systematic review and meta-analysis of correlation and intervention studies
Source: Psychol Med. 2021 Apr 8;51(5):716–26. doi: 10.1017/S0033291721000854 (PMC8108391; doi:10.1017/S0033291721000854)
Supplement: Supplementary file 1 [file S0033291721000854sup.zip › S0033291721000854sup001.docx]

# Supplemental Material

# Appendix S1: Search Strategy and Data Extraction

## **Search Strategy**

The research question was specified by using the PICO-method (Aslam & Emmanuel, 2010): “In different samples (P), what associations exist between continuum beliefs of mental health and mental illness (I) and stigmatization of persons with mental illnesses (O) (as compared to seeing mental health and mental illness as distinct categories (C))?”

- *Continuum beliefs:* continuum belief* OR continuity belief* OR continu*
- *Stigmatization:* stigma* OR attitude* OR stereotype* OR discrimination
- *Mental illness:* “mental illness*” OR “mental health” OR “mental disorder*” OR “psychiatric illness” OR “psychiatric health” OR depress* OR schizophrenia OR alcohol* OR “substance use” OR “substance abuse” OR bipolar OR “obsessive compulsive” OR anxiety OR “eating disorder” OR “post-traumatic stress disorder” OR “PTSD”
- *Combined by Boolean Operators:* ((((continuum belief* OR continuity belief* OR continu*))) AND ((stigma* OR attitude* OR stereotype* OR discrimination))) AND ((“mental illness*” OR “mental health” OR “mental disorder*” OR “psychiatric illness” OR “psychiatric health” OR depress* OR schizophrenia OR alcohol* OR “substance use” OR “substance abuse” OR bipolar OR “obsessive compulsive” OR anxiety OR “eating disorder” OR “post-traumatic stress disorder” OR “PTSD”))

## **Data extraction and coding**

study-level: year of publication, country, sample population, statistical analysis, measurement, scale range of predictor and outcome, general findings and conclusions

sample-level: sample size, mean age, type of mental disorder

effect size-level: means, standard deviations, unstandardized and standardized coefficients, confidence intervals, correlation coefficients, p-values (Coding on effect size-level was only done for inclusion into meta-analysis)
