# Supplementary material for: Continuum beliefs and mental illness stigma: a systematic review and meta-analysis of correlation and intervention studies
Source: Psychol Med. 2021 Apr 8;51(5):716–26. doi: 10.1017/S0033291721000854 (PMC8108391; doi:10.1017/S0033291721000854)
Supplement: Supplementary file 1 [file S0033291721000854sup.zip › S0033291721000854sup002.docx]

# Appendix S2: Search Strategy

### Quality Assessment – Modified version of Downs and Black Quality Assessment

The item 27 regarding the power of the studies was modified to facilitate use. Studies with subgroups of N ≥ 250 have been rated with 1. Most of the studies are cross-sectional group comparisons without follow-ups. Therefore, some items (e.g. regarding follow-up) of Downs and Black Quality Assessment (27) are irrelevant for the studies, included in the systematic review. Some items have been left out completely, while other items are only relevant for some of the studies. The varying number of rated items for the studies should be considered.

Table S2.1. Modified version of Downs and Black Quality Assessment. 27 items of Downs and Black Quality Assessment* and modification/ not considered items

|  | **Item** | **Modification** |
| --- | --- | --- |
| 1 | Is the hypothesis/aim/objective of the study clearly described? |  |
| 2 | Are the main outcomes to be measured clearly described in the Introduction or Methods section? |  |
| 3 | Are the characteristics of the patients included in the study clearly described? |  |
| 4 | Are the interventions of interest clearly described? |  |
| 5 | Are the distributions of principal confounders in each group of subjects to be compared clearly described? |  |
| 6 | Are the main findings of the study clearly described? |  |
| 7 | Does the study provide estimates of the random variability in the data for the main outcomes? |  |
| 8 | Have all important adverse events that may be a consequence of the intervention been reported? | not considered |
| 9 | Have the characteristics of patients lost to follow-up been described? |  |
| 10 | Have actual probability values been reported for the main outcomes except where probability value is less than 0.001? |  |
| 11 | Were the subjects asked to participate in the study representative of the entire population from which they were recruited? |  |
| 12 | Were those subjects who were prepared to participate representative of the entire population from which they were recruited? |  |
| 13 | Were the staff, places, and facilities where the patients were treated, representative of the treatment of the majority of patients receive? | not considered |
| 14 | Was an attempt made to blind study subjects to the intervention they have received? |  |
| 15 | Was an attempt made to blind those measuring the main outcomes of the intervention? | not considered |
| 16 | If any of the results of the study were based on "data dredging", was this made clear? |  |
| 17 | In trials and cohort studies, do the analyses adjust for different lengths of follow-up of patients, or is the time period between the intervention and outcome the same for cases and controls? |  |
| 18 | Were the statistical tests used to assess the main outcomes appropriate? |  |
| 19 | Was compliance with the intervention reliable? |  |
| 20 | Were the main outcome measures used accurate (valid and reliable)? |  |
| 21 | Were the patients in different intervention groups resp cases/controls recruited from the same population? |  |
| 22 | Were study subjects in different intervention groups resp cases/controls recruited over the same period of time? |  |
| 23 | Were study subjects randomized to intervention groups? |  |
| 24 | Was the randomized intervention assignment concealed from both patients and health care staff until recruitment was complete and irrevocable? | not considered |
| 25 | Was there adequate adjustment for confounding in the analyses from which the main findings were drawn? |  |
| 26 | Were losses of patients to follow-up taken into account? |  |
| 27 | Did the study have sufficient power to detect a clinically important effect where to probability value for a difference being due to chance is less than 5%? | 1: N ≥ 250 per subgroup; 0: N≤ 250 per subgroup |

Table S2.2. Quality Assessment of all included studies using Modified version of Downs and Black Quality Assessment.

| **ID** | **Study** | **1** | **2** | **3** | **4** | **5** | **6** | **7** | **9** | **10** | **11** | **12** | **14** | **16** | **17** | **18** | **19** | **20** | **21** | **22** | **23** | **25** | **26** | **27** | **Sum** | **%** |
| --- | --- | --- | --- | --- | --- | --- | --- | --- | --- | --- | --- | --- | --- | --- | --- | --- | --- | --- | --- | --- | --- | --- | --- | --- | --- | --- |
| 2 | Angermeyer et al., 2015 | 1 | 1 | 1 | - | 0 | 1 | 0 | - | 0 | 0 | 0 | 1 | 1 | - | 1 | 0 | 1 | 1 | 1 | 1 | 1 | - | 1 | 13 | 61,90 |
| 3 | Cassidy & Erdal, 2019 | 1 | 1 | 1 | 1 | 0 | 1 | 1 | - | 1 | 0 | 0 | 1 | 1 | - | 1 | 1 | 1 | 1 | 1 | 1 | 0 | - | 0 | 15 | 71,43 |
| 4 | Corrigan et al., 2015 | 1 | 1 | 1 | - | 2 | 1 | 1 | - | 0 | 0 | 0 | - | 1 | - | 1 | 1 | 1 | 1 | 1 | - | 0 | - | 1 | 14 | 82,35 |
| 5 | von dem Knesebeck et al., 2015 | 1 | 1 | 1 | 1 | 2 | 1 | 1 | - | 1 | 1 | 1 | 1 | 1 | - | 1 | 0 | 1 | 1 | 0 | 0 | 1 | - | 1 | 18 | 90,00 |
| 6 | Makowski et al., 2016a | 1 | 1 | 1 | 1 | 2 | 1 | 1 | - | 0 | 1 | 1 | 1 | 1 | - | 1 | 0 | 1 | 1 | 1 | 0 | 1 | - | 1 | 18 | 90,00 |
| 7 | Schlier & Linoln, 2019 (study 3) | 1 | 1 | 1 | 1 | 1 | 1 | 1 | - | 1 | 0 | 0 | 1 | 1 | - | 1 | 1 | 1 | 1 | 1 | 1 | 1 | - | 0 | 17 | 85,00 |
| 8 | Schlier & Scheunemann, 2016 (study 2) | 1 | 1 | 1 | - | 2 | 0 | 0 | - | 0 | 0 | 1 | - | 1 | - | 1 | 0 | 1 | 1 | - | - | 0 | - | 1 | 11 | 64,71 |
| 10 | Schomerus et al., 2013 | 1 | 1 | 1 | 1 | 1 | 1 | 0 | - | 1 | 1 | 1 | 1 | 1 | - | 1 | 0 | 1 | 1 | 1 | 1 | 1 | - | 1 | 18 | 90,00 |
| 11 | Schomerus et al., 2015 | 1 | 1 | 1 | 1 | 0 | 1 | 1 | - | 0 | 0 | 0 | 1 | 1 | - | 1 | 0 | 1 | 1 | 0 | 0 | 0 | - | 1 | 12 | 60,00 |
| 12 | Speerforck et al., 2019 | 1 | 1 | 1 | 1 | 1 | 1 | 1 | - | 1 | 1 | 1 | 1 | 1 | - | 1 | 1 | 1 | 1 | 1 | 1 | 1 | - | 1 | 20 | 100,00 |
| 13 | Subramaniam et al., 2017 | 1 | 1 | 1 | 1 | 0 | 1 | 1 | - | 1 | 0 | 1 | 1 | 1 | - | 1 | 0 | 1 | 1 | 1 | 1 | 1 | - | 1 | 17 | 85,00 |
| 14 | Wiesjahn et al., 2014 | 1 | 1 | 1 | - | 1 | 0 | 1 | - | 0 | 0 | 0 | - | 1 | - | 1 | 1 | 1 | 1 | 1 | - | 1 | - | 1 | 13 | 76,47 |
| 15 | Clement et al., 2010? | 1 | 1 | 1 | 1 | 0 | 1 | 1 | - | 1 | 0 | 0 | - | 1 | - | 1 | 1 | - | 1 | 1 | - | 0 | - | 1 | 13 | 76,47 |
| 16 | Cole & Warman, 2019 | 1 | 1 | 1 | 1 | 2 | 1 | 1 | - | 1 | 0 | 0 | 1 | 1 | - | 1 | 1 | 1 | 1 | 1 | 1 | 1 | - | 0 | 18 | 90,00 |
| 17 | Corrigan et al., 2017 | 1 | 1 | 1 | 1 | 2 | 1 | 0 | - | 0 | 0 | 0 | 1 | 1 | - | 1 | 1 | 1 | 1 | 1 | 1 | 1 | - | 1 | 17 | 85,00 |
| 18 | Cumming & Cumming, 1957 | 1 | - | 1 | 1 | 2 | 1 | 0 | - | 0 | 0 | 0 | 1 | 1 | - | 0 | 0 | 0 | 0 | 0 | 0 | 0 | - | 1 | 9 | 47,37 |
| 19 | Dobson et al., 2019 | 1 | 1 | 1 | 1 | 1 | 1 | 1 | 0 | 1 | 0 | 0 | - | 1 | 1 | 1 | 0 | 1 | - | - | - | 1 | 0 | 1 | 14 | 73,68 |
| 20 | Dolphin & Hennessy, 2017 | 1 | 1 | 1 | 1 | 0 | 1 | 1 | - | 1 | 0 | 0 | 1 | 1 | - | 1 | 1 | 1 | 1 | 1 | 0 | 1 | - | 1 | 16 | 80,00 |
| 21 | Helmus et al., 2019 | 1 | 1 | 0 | 1 | 1 | 1 | 1 | 1 | 1 | 0 | 0 | 1 | 1 | 1 | 1 | 1 | 1 | 1 | 1 | 1 | 0 | 0 | 0 | 17 | 73,91 |
| 22 | Morris et al., 2020 | 1 | 1 | 1 | 1 | 0 | 1 | 1 | - | 1 | 0 | 0 | 1 | 1 | - | 1 | 1 | 1 | 1 | 1 | 1 | 1 | - | 1 | 17 | 80,95 |
| 23 | Makowski et al., 2016b | 1 | 1 | 1 | 1 | 2 | 1 | 1 | - | 1 | 1 | 1 | 0 | 1 | - | 1 | 1 | 1 | 1 | 1 | 0 | 1 | - | 1 | 19 | 95,00 |
| 24 | Schomerus et al., 2016 | 1 | 1 | 1 | 1 | 2 | 0 | 0 | - | 1 | 1 | 1 | 1 | 1 | - | 1 | 1 | 1 | 1 | 1 | 1 | 1 | - | 1 | 19 | 95,00 |
| 25 | Schulze et al., 2003 | 1 | 1 | 1 | 1 | 2 | 1 | 1 | 0 | 1 | 0 | 0 | 0 | 1 | 1 | 1 | 0 | 1 | - | 1 | 0 | 1 | 0 | 0 | 15 | 68,18 |
| 26 | Shiraishi et al., 2019 | 1 | 1 | 1 | 1 | 2 | 1 | 1 | 1 | 1 | 0 | 0 | 0 | 1 | 1 | 1 | 1 | 1 | 1 | 1 | 1 | 1 | 1 | 1 | 21 | 91,30 |
| 27 | Szeto et al., 2019 | 1 | 1 | 1 | 1 | 1 | 1 | 1 | 1 | 1 | 0 | 0 | - | 1 | 1 | 1 | 0 | 1 | - | - | - | 1 | 0 | 1 | 15 | 71,43 |
| 28 | Thibodeau, 2017 | 1 | 1 | 0 | 1 | 0 | 0 | 0 | - | 0 | 0 | 0 | 1 | 1 | - | 1 | 1 | 1 | 1 | 1 | 1 | 0 | - | 0 | 11 | 55,00 |
| 29 | Thibodeau et al., 2018 | 1 | 1 | 1 | 1 | 0 | 1 | 1 | - | 0 | 0 | 0 | 1 | 1 | - | 1 | 1 | 1 | 1 | 1 | 1 | 0 | - | 0 | 14 | 70,00 |
| 30 | Thibodeau & Peterson, 2018 | 1 | 1 | 1 | 1 | 0 | 1 | 1 | - | 1 | 0 | 0 | 1 | 1 | - | 1 | 1 | 1 | 1 | 1 | 1 | 1 | - | 0 | 16 | 80,00 |
| 31 | Thibodeau et al., 2019 | 1 | 1 | 1 | 1 | 0 | 1 | 0 | - | 1 | 0 | 0 | 1 | 1 | - | 1 | 1 | 1 | 1 | 1 | 1 | 1 | - | 1 | 16 | 80,00 |
| 32 | Wiesjahn et al., 2016 | 1 | 1 | 1 | 1 | 2 | 1 | 1 | - | 0 | 0 | 0 | 1 | 1 | - | 1 | 1 | 1 | 1 | 1 | 1 | 1 | - | 1 | 18 | 90,00 |
| 33 | Violeau et al., 2020 | 1 | 1 | 1 | 1 | 0 | 1 | 1 | - | 1 | 0 | 0 | 1 | 1 | - | 1 | 1 | 1 | 1 | 1 | 1 | 0 | - | 0 | 15 | 75,00 |

*Annotations: Downs and Black Quality Assessment: Downs SH, Black N. The feasibility of creating a checklist for the assessment of the methodological quality both of randomised and non-randomised studies of health care interventions. J Epidemiol Community Health 1998;52:377–384. 23 items, Item number according to original scale. Sum score (Sum) and Percent (%) of 1-rates in relation to possible score.*
