# Supplementary material for: Continuum beliefs and mental illness stigma: a systematic review and meta-analysis of correlation and intervention studies
Source: Psychol Med. 2021 Apr 8;51(5):716–26. doi: 10.1017/S0033291721000854 (PMC8108391; doi:10.1017/S0033291721000854)
Supplement: Supplementary file 1 [file S0033291721000854sup.zip › S0033291721000854sup003.docx]

# Appendix S3: Overview of selected studies

Table S3.1. Overview of selected studies investigating continuum beliefs (CB), mental illness and stigma

| **Authors, year** | **Country** | **Sample** | **Methodology** | **Measurement of CB** | **Analysis** | **Topic/ mental disorder** | **Findings** |
| --- | --- | --- | --- | --- | --- | --- | --- |
| Alvarado Chavarria, 2013 | US | N = 10, activists with own experiences | Qualitative study;  semi-structured interview, coding of answers | CB in the context of Link and Phelan’s Labeling component | iterative coding approach | Perspectives on Link & Phelan model of stigma (2001) | One of the emergent themes: “focus on a shared continuum of human experience”: challenging the borders of normal/abnormal, “differences based on degrees but not absolute differences” (p. 94) CB as a part of the stigma model inherent to labeling component |
| Anger-meyer et al., 2015 | France | N = 1600, general population | Cross-sectional study;  vignettes (depression or schizophrenia) | One-Item Measure (Schomerus et al., 2013) | Multiple regression analyses | Depression; Schizophrenia | CB associated with less desire for social distance (B= ‑0.18*(depr.) / ‑0.23*(schiz.)) and more Pro-social reactions (B = 0.25* (depr.) / 0.18* (schiz.)); no sign. association with fear, association with anger only for schiz.(B = 0.11*); results did not differ sign. between disorders |
| Cassidy & Erdal, 2019 | US | N = 377 (Main Study) | Cross-sectional study;  vignettes;  CB manipulation: 2 causes (biological, psycho-social) x 3 treatments (biogenetic, psycho-social, combined)) | Continuity with Normal Experience subscale (Norman et al., 2012) | 2 x 3 ANOVA | Bipolar disorder | Bipolar disorder was viewed as less continuous with normal experience when explained biogenetically, F(1,221)=4.87, p=.028, *η^2^* =.022, association between CB and stigma not directly tested |
| Clement et al., 2010 | UK (inter-national partici- pants) | N = 32, mental health experts at International Stigma Conference | Qualitative study;  ratings of message types to include in population-level campaigns (1. Rating, discussion in group, 2. rating); | Experts rated 10 different components  of anti-stigma messages, CB was one of the components | Wilcoxon and Mann-Whitney tests | Mental health in general | CB as part of messages that counter the "otherness", positive personal experiences of the use of CB messages; all in all CB is not one of the recommended messages for anti-stigma campaigns, with high changes in consensus levels from rating 1 to rating 2; some similar messages that are recommended: "see the person", "social inclusion/human rights", messages depending on particular audience |
| Cole & Warman, 2019 | US | N = 178, general population | Longitudinal study (pre/ post); vignettes;  CB Manipulation: 3 explanations for mental state (biogenetic, continuum, control) | “People who have OCD have symptoms that are similar to the occasional experiences of ordinary people.” (Thibodeau, 2017) | 3 x 2 ANOVA | OCD with violent intrusive thoughts | Manipulation check (more CB reported in CB condition); reduction in social distance for CB condition (*M_pre_*=16.25, *M_post_*=15.02, t=3.53, *p*=.001); same effect for biogenetic explanations, both greater reduction than control group; sig. reduction of dangerousness for CB condition (*M_pre_*=25.39, *M_post_*=23.29, t=3.10, *p*=.003), more than for biogenetic but no sign.; ns. for blame |
| Corrigan et al., 2015 | US | N = 460, general population | Cross-sectional study; Comparison of stigma-assessment measures, vignettes | Semantic Differential Scale (Measures of differences) | ANOVA, Multiple regression analyses | Schizophrenia | Sign. Differences between stigma measures, mental illness descriptors were sign. associated with all three indices of difference(*r* ranged from 0.24–0.50, *p*=.001), Semantic Differential, Similar-Difference: B=0.29*, highest stigmatizing scores, greatest endorsements, significantly higher than the two other difference scores, confirmation of principle correlation of CB and stigma |
| Corrigan et al., 2017 | US | N = 598, general population | Cross-sectional study; vignettes;  CB Manipulation: 2 messages (continuum, categorical, neutral) x 2 processes (contact (videotape), education (text presentation)) | CBQ (Wiesjahn et al., 2014) | 3 x 2 ANOVA | Schizophrenia | Manipulation check (more CB reported in CB condition);  Stigmatizing Beliefs (AQ-8): n.s.; Difference Scale: improvement for continuum message for those in the contact (F(2,296) = 5.96, p<.005) but not the education condition (ns.); the continuum message yielded significantly lower (p<.05) difference scores than those in the categorical or neutral conditions. |
| Cumming & Cumming, 1957 | Canada (Blackfoot) | N = 540, general population | Longitudinal study (pre/post) in intervention and control region;  Intervention: 6-month awareness program including film festival, radio program, school coop. | No CB measure, but CB included in intervention (1 of 3 working principles: “There is a continuum between normality and abnormality”) |  | General beliefs about mental illness and mental health | Before and after the program: no changes on measures of social responsibility scale and social distance scale (no direct measure of CB or associations of CB and stigma) |
| Dobson  et al., 2019 | Canada | N = 1155, workplace | Longitudinal study (3 time points), Intervention for workplace-setting: The working mind program to reduce stigma using a continuum model with 2 versions (4h for frontline workers, 8h for managers); “train-the-trainer” model | CB included in intervention; “Have You Used Any of What You Learned at The Working Mind at Work or at Home: Mental health continuum model (self and/or others)” | Study-level meta-analysis | Workplace mental health | Lower baseline stigma scores for participants who completed all 3 assessments, sign. pre- to post-change on the workplace attitudes scale, sig. reductions in stigma for the total scale, coefficient=0.167, *SE*=0.08, *z*=20.72,  *p*<.001; Question about CB Use: 21.3% of cases answered yes (n=57) |
| Dolphin & Hennessy, 2017 | Ireland | N = 156, adolescents | Cross-sectional study; audio-visual vignette characters with varying depressive symptom severity (impact of depression labels);  CB manipulation: 3 conditions (control, label, continuum) | Version of Schomerus et al., 2013: “We are all sometimes like Simon or Killian, it’s just a question of how extreme that state is” | 2 x 3 x 3 mixed between-within subjects ANOVAs | Depression | Manipulation check (CB group endorsed CB more);  no sign. effects of label or CB on emotional reactions, sign. interaction for within category assimilation (WCA) sympathy scores; for WCA anger scores sig. main effect for condition, F(2, 150)=5.44, *p*=.005, regardless of time and gender, the continuum group had sig. higher scores; no effect of labeling |
| Helmus et al., 2019 | Netherlands | N = 202, mental health professionals | Longitudinal study (2 time points); 2 conditions (experimental, control), 2h-Workshop (various elements: education, sharing experiences, cognitive interventions on stigma; aim: decreasing stigma, stimulating CB) | CBQ (Wiesjahn et al., 2014) | 2 x 2 ANOVA | Beliefs of health care providers | Manipulation check (more CB reported in CB condition),  CB mean score before the workshop (*M*=4.39, *SD*=.46) sign. lower than the after the workshop (*M*=4.63, *SD*=.46; t(46)=−4.60, *p*<.01); no sign. effect on stigmatizing attitudes (possible explanation: social desirability because of obvious aim of intervention) |
| Makowski et al., 2016a | Germany | N = 1338 (group 1),  N = 1316 (group 2); general population | Cross-sectional study; vignettes,  telephone survey | One-Item Measure (Schomerus et al., 2013) | Path models | Depression & Schizophrenia | Less desire for social distance (B=-0.14* (depr.)/ -0.13*(schiz.)); greater pro-social reactions (B=0.09* (depr.)/ 0.07* (schiz.)); no sign. associations between CB and fear, anger, and stereotypes |
| Makowski et al., 2016b | Germany | N = 2014 (pre);  N = 2006 (post),  general population | Longitudinal study (pre/ post) in city with mental health awareness campaign psychenet and control region; vignettes | No CB measure, but CB included in intervention (“Yes, it can hit virtually anybody.”) | 2 x 2 ANOVAs | Depression & Schizophrenia | General Public: no significant effects on desire for social distance and emotional reactions, People with awareness of the campaign: reduced desire for social distance (F(1,659)=8.89; *p*=.003) for depression vignette; sign attribution of “in need of help” (F(1,666)=7.48; *p*=0.006) for schizophrenia vignette |
| Morris  et al., 2020 | UK | N = 597, general population with addiction experience (alcohol use) | Cross-sectional study;  video vignettes  CB manipulation: 3 explanations (continuum, binary disease model (BDM), control) | Problem Drinking Belief Scale | 3 x 2 x 2 between participants factorial ANCOVA | Alcohol use disorder, Harmful drinking | Manipulation check (sig. more problem framing for CB vs. BDM),  CB associated with higher problem recognition amongst harmful drinkers with no addiction experience; sig. main effect of condition (F(2,189)=4.15, *p*=.017), problem recognition was sig. higher in the CB condition compared to the BDM condition (*p*=.014); no direct association between CB and stigma tested |
| Schlier & Lincoln, 2019 (study 3) | Germany | N = 137, general population | Cross-sectional study;  Who-said-what-task: to allocate statements to individuals who belong to different social groups (e.g., schizophrenia /depression vs. healthy) | Brief assessment of CB (rating target person as similar to oneself or to others) after Who Said What-task | Multiple regression analyses | Schizophrenia & Depression vs. Healthy | No sign. association between a person's WSW-score and self-reported perceived similarity of target persons with mental illness label to others (b=−0.04) and to oneself (b=−0.03); suggested reason: divergence between self-reported measures and implicit associations |
| Schlier  et al., 2016 (study 2) | Germany | N = 363, general population | cross-sectional study;  CBQ-R scale construction (item selection and correlations with related constructs) | CBQ-R | Correlations analyses | Schizophrenia | CB is sign. correlated with less social distance (*r*= ‑.25), less perceived dangerousness (*r*= ‑.22), less unpredictability (*r*= ‑.24), but not sign. with attribution of responsibility (*r*= ‑0.09) |
| Schoech, 2017 | US | N = 8; Participants with (n = 4) and without (n = 4) experience of working with people with intellectual disabilities and PTSD | Qualitative phenomenological study; semi structured open-ended recorded interviews | interview question: to “describe a person with intellectual disability”, “where they live and what they do” |  | Trauma and posttraumatic stress disorder (PTSD) of people with intellectual disabilities | 8 themes identified through interviews, one is: “they’re just folks like us”: words used like, “wide range or spectrum of abilities, “You know, I think everyone could be considered to have a disability because we all have struggles, you know, none of us are 100% at everything. And so that is kind of how I go about doing it, is that we all have disability-ties, in the fact that, whether they are medical, behavioral, or mental.” (p. 149); association between CB and stigma not directly tested |
| Schomerus et al., 2013 | Germany | N = 3642, general population | cross-sectional study;  vignettes,  fully structured interview | ‘‘Basically we are all sometimes like this person. It’s just a question how pronounced this state is.’’ (original) | Linear regression analyses | Schizophrenia Depression Alcohol Use Disorder | CB associated with less desire for social distance (B = ‑0.16* (depr.)/ ‑0.31* (schiz.)/ ‑0.21* (alc.)) and more pro-social reactions (B = 0.19* (depr.)/ 0.30* (schiz.)/ 0.22* (alc.)); sign. less fear and more anger for depr. and schiz., largest effects for schiz., smallest effects for depr. |
| Schomerus et al., 2015 | Germany | N = 598 (2014),  N = 806 (2015), general population | Longitudinal study (2 time points before and after German Wings plane crash),  vignettes | One-Item Measure (Schomerus et al., 2013) | Logit regression model | Depression (after plane crash), Schizophrenia | CB slightly decreased (22% vs. 27% CB), no sig. changes in emotional reactions and desire for social distance, association between CB and stigma not directly tested |
| Schomerus et al., 2016 | Germany | N = 1679, general population | Cross-sectional study;  vignette about schizophrenia or depression  CB manipulation: bogus newspaper articles with information (continuum, dichotomy, control), | 7 items on continuum and differentness beliefs (e.g. ‘‘There is something about Anne that makes her fundamentally different from other people’’) | Multiple linear regression; linear structural equation models | Schizophrenia, Depression | Manipulation check (more CB reported in CB condition),  lower fundamental difference (*r*= ‑0.29*), stronger social acceptance (*r*= 0.25*), lower unpredictability (*r*= ‑0.30*), more blame (*r*= 0.06*); group differences: CB: reduced perceived fundamental difference (adjusted model, ‑0.19 SD, *p*=.001), increased social acceptance (0.18 SD, *p*=.003), reduced blame (‑0.18 SD, *p*=.05); partial mediation of CB on stigma by CB condition: 24.9% (CI: 6.3; 43.4) of the total effect of CB on perceived difference and 26.4% (CI: 5.9; 46.8) of total effect on social acceptance mediated by CB |
| Schulze et al., 2003 | Germany | Intervention: N = 90 control:  N = 60, adolescents | Longitudinal study (pre/ post);  Intervention: School project “Crazy? So, what! – It’s normal to be different”, getting to know a person with schizophrenia, focusing on similarities rather than differences | No CB measure, but CB included in intervention | Two-level random coefficient regression model;  cross-level interaction effects | Schizophrenia | Attitude improvement only for unsure responses (to positive), positive effects on stigmatization and social distance through project still existent in 1-month follow-up; intervention: sig. stigma reduction, readiness to enter relationships as expected long-term process |
| Shiraishi et al., 2019 | Japan | N = 74, caregivers | Longitudinal study (3 time points);  Intervention: educational program based on a normalizing approach (continuum of psychotic-like experiences and healthy individuals); combined with TAU | No CB measure, but CB included in intervention | ANCOVA | Caregivers of patients with schizophrenia | Links Stigma Scale: no sign. difference in stigma after 10 weeks and 14 weeks, adjusted for baseline group differences |
| Speerforck et al., 2019 | Germany | N = 1008, general population | cross-sectional study; vignettes (child/adult) | One-Item Measure (Schomerus et al., 2013), additional item (Normality) | Path models | ADHD  (child, adult) | Social distance: B= -0.18* (adult); pro-social reactions B= 0.21* (child)/ 0.20* (adult); abnormality: anger: B= 0.15* (child), fear:  B= 0.16* (child) |
| Subramaniam et al., 2017 | Singapore | N = 3006, general population | Cross-sectional study; vignettes, structured  face-to-face interviews | One-Item Measure (Schomerus et al., 2013) | Multiple linear regression analyses | Alcohol use disorder, Dementia, Depression, Schizophrenia, OCD | Social distance (supplementary material):  B= 0.04 n.s. (depr.)/ ‑0.14* (schiz.)/ ‑0.2* (alc.)/ -0.14* (OCD); CB is sig. related to ‘weak-not-sick’ thinking for dementia and schizophrenia; CB and dangerousness/unpredictability n.s. |
| Szeto et al., 2019 | Canada | N = 4649,  16 sites, police, first responder (police, fire – or emergency services, paramedics) | Longitudinal study (pre/ post);  Intervention: anti-stigma program “Road to Mental Readiness”  (components: stigma reduction through video contact-based education, Mental Health Continuum Model, coping and resilience skills) | CB included in intervention; “Have You Used Any of What You Learned at The R2MR at Work or at Home: Mental health continuum model (self and/or others)” | Meta-analysis | General beliefs about mental health and mental illness | Sig. stigma reduction after the program  (workplace attitudes scale: Social distance/ avoidance, dangerousness/ unpredictably, Work Beliefs, helping behavior, responsibility for Illness), overall mean stigma reduction of 0.12 scale points pre to post; overall workplace attitudes scale n.s. for post to follow up; positive statements for use of continuum model after program: 12.5% of cases (n=65) |
| Thibodeau, 2017 | US | N = 308, general population | Cross-sectional study;  vignettes;  CB manipulation: research summary (continuum, categorical, no text control) | 1 item continuum: “People who have schizophrenia have symptoms [delusions, hallucinations] that are similar to the occasional experiences of ordinary people”  1 item categorical: “People who have schizophrenia are fundamentally different from ordinary people”; | Correlations, MANOVA | Schizophrenia | Manipulation check (more CB endorsement in CB condition);  CB: lower desire for social distance, lower unpredictability, and marginally less fear. categorical: more desire for social distance; more fear; n.s. effects of the CB manipulation on stigma (*λ*=0.98, F(12, 600)=0.49, *p*>.05, *ηp²*=.011) |
| Thibodeau & Peterson, 2018 | US | N = 135, under-graduates | Longitudinal study (pre/post);  CB manipulation: bogus interview (continuum, categorical, control) and bogus scientific articles (continuum, categorical, control);  lexical decision task to assess momentary affect | CB 4-item scale based on Thibodeau, 2017, 2 items CB, 2 items categorical | Correlations, ANOVA | Schizophrenia | Manipulation check (more CB endorsement in CB condition);  CB is related to lower desire for social distance, less pro-social reactions, weaker stereotype attitudes in correlational analyses but no significant difference between conditions concerning stigma outcomes;  momentary affect: CB group with faster RTs (F(1,117)=5.73, p=.018,*ηp^2^*=0.05) to anxiety words (interpreted as higher sense of threat), CB group also with greater self-reported fear |
| Thibodeau et al., 2018 | US | N = 69, under-graduates | Cross-sectional study;  subjects were given information about a man (describing schizophrenia) that they would have to interview later followed by seat selection task;  CB manipulation: bogus scientific article (continuum, categorical, control) plus follow up task to boost manipulation; | CB 4-item scale based on Thibodeau, 2017: 2 items CB, 2 items categorical | Correlations, ANOVA | Schizophrenia | Manipulation check (more CB endorsement in CB condition);  CB manipulation was related to less desire for social distance than control (marginally) and categorical (F(1,66)=5.37, *p*<.03, *ηp²*=.08); less pro-social reactions than categorical (F(1,66)=4.93, *p*<.03, *ηp^2^*=.07): categorical group showed greater endorsement of dangerousness (F(1,66)=4.95, *p*<.03, *ηp^2^*=.07) and unpredictability (F(1,64)=7.14, *p*<.01, *ηp^2^*=.10) than CB (and control); n.s. for fear and anger; seat selection task: CB manipulation did not affect initial or final seat selection; |
| Thibodeau, 2019 | US | N = 654, general population, self-reported depression | Longitudinal study (pre/post);  CB manipulation: short magazine article about depression with bogus scientific article (continuum, categorial, control) | 6-item scale (based on Thibodeau & Peterson, 2018) | Correlations, ANOVA | Depression | Manipulation check (more CB endorsement in CB condition);  generally CB related to a decreased desire for social distance, marginally less anger, more pro-social reactions; general categorical beliefs are related to increased desire for social distance, anger, stigma agreement; n.s. effects of CB manipulation on stigma variables (general population and self-reported depression) |
| Violeau  et al., 2020 | France | N = 565, general  population | Cross-sectional study;  vignette videos;  CB manipulation: 3 explanations (continuum, categorical, neutral) | Questionnaire  of Belief in a Continuum in Schizophrenia (QBCS, 4 items), adaption of Wiesjahn et al., 2014 | ANOVA | Schizophrenia | Manipulation check (no differences in CB endorsement in CB condition compared to control group);  CB and categorical as rather independent, significant mediations of the association between CB/categorical beliefs and essentialism and self-stereotype by perceived similarities |
| Von dem Knesebeck et al., 2015 | Germany | N = 650 (2014),  N = 601 (2015), general population | Longitudinal study (2 time points, before and after German Wings plane crash),  telephone surveys with vignettes | One-Item Measure (Schomerus et al., 2013) | ANOVA | Depression | Sign. higher stigma after plane crash:  CB decreased (F(1,1231)=9.92, *p*=.002), more unpredictability, dangerousness, fear, anger; correlation between CB and stigma not directly tested |
| Wiesjahn et al., 2014 | Germany | N = 120, general population | Cross-sectional study;  online-survey | CBQ: 16-item Continuum Beliefs Questionnaire (original) | Correlations, hierarchical multiple regression analysis | Schizophrenia | Stereotypes: *r*= -0.19*, social distance:  *r*= -0.15 (ns); CB explained a sign. amount of variance in stereotypes; CB explained a significant additional amount of variance in stereotypes: B= -0.20* |
| Wiesjahn et al., 2016 | Germany | N = 1189, general  population | Longitudinal study (pre/post);  vignettes;  CB manipulation: 3 different information texts (continuum, biogenetic, neutral) | CBQ (Wiesjahn et al., 2014) | MANCOVA | Schizophrenia | Manipulation check (more CB reported in CB condition);  CB showed correlations with stigma variables (social distance: *r*= ‑.35*, dangerousness: *r*= ‑.26*; responsibility: *r*= ‑.10*; fear: *r*= ‑.20*) CB condition showed sign. less incompetence/unpredictability, biogenetic condition showed less responsibility/blame than CB |

Annotations: * = p < .05, n.s. = non-significant
