# Supplementary material for: Continuum beliefs and mental illness stigma: a systematic review and meta-analysis of correlation and intervention studies
Source: Psychol Med. 2021 Apr 8;51(5):716–26. doi: 10.1017/S0033291721000854 (PMC8108391; doi:10.1017/S0033291721000854)
Supplement: Supplementary file 1 [file S0033291721000854sup.zip › S0033291721000854sup004.docx]

# Appendix S4: Meta-Analyses of continuum beliefs

Table S4.1. Meta-analysis and forest plot of the association of continuum beliefs and pro-social reactions (single study effect-sizes and combined effect-size).

|  | **Study** | **Disorder** | ***r*** | **L CI** | **U CI** | **Weight** | 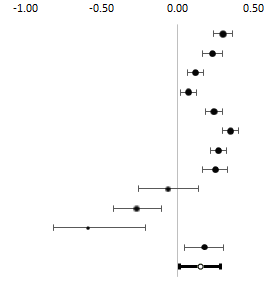 |
| --- | --- | --- | --- | --- | --- | --- | --- |
| 1 | Angermeyer et al., 2015^1^ | Depr. | 0.30 | 0.24 | 0.36 | 9.44% |  |
| 2 | Angermeyer et al., 2015^1^ | Schiz. | 0.23 | 0.16 | 0.29 | 9.44% |  |
| 3 | Makowski et al., 2016a^1^ | Depr. | 0.12 | 0.06 | 0.17 | 9.72% |  |
| 4 | Makowski et al., 2016a^1^ | Schiz. | 0.07 | 0.02 | 0.13 | 9.72% |  |
| 5 | Schomerus et al., 2013^1^ | Depr. | 0.24 | 0.19 | 0.29 | 9.67% |  |
| 6 | Schomerus et al., 2013^1^ | Schiz. | 0.35 | 0.30 | 0.40 | 9.67% |  |
| 7 | Schomerus et al., 2013^1^ | Alc. | 0.27 | 0.22 | 0.32 | 9.65% |  |
| 8 | Speerforck et al., 2019^1^ | ADHD | 0.25 | 0.17 | 0.33 | 9.04% |  |
| 9 | Thibodeau, 2017^1^ | Schiz. | -0.06 | -0.25 | 0.14 | 6.29% |  |
| 10 | Thibodeau & Peterson, 2018^2^ | Schiz. | -0.27 | -0.42 | -0.10 | 6.94% |  |
| 11 | Thibodeau et al., 2018^2^ | Schiz. | -0.59 | -0.81 | -0.21 | 2.49% | 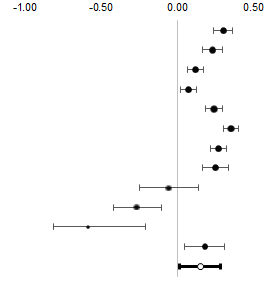 |
| 12 | Thibodeau, 2019^1^ | Depr. | 0.18 | 0.05 | 0.31 | 7.92% |  |
|  |  |  | **0.15** | **0.01** | **0.28** |  |  |

Annotations: Population: ^1^ = General population, ^2^ = Undergraduates; Disorder: Depr = Depression, Schiz.= Schizophrenia, Alc = Alcoholism, ADHD = Attention Deficit/Hyperactivity Disorder, OCD = Obsessive-compulsive disorder); r = correlation coefficient; L CI/ U CI = lower and upper limit of confidence interval. Weight: Study weight. Forest Plot: single study effect-sizes and combined effect-size with CI. Size of point reflects study weight.

Table S4.2. Meta-analysis and forest plot of the association of continuum beliefs and fear (single study effect-sizes and combined effect-size).

|  | **Study** | **Disorder** | ***r*** | **L CI** | **U CI** | **Weight** | **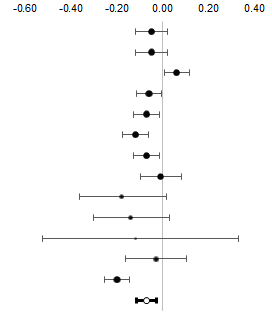** |
| --- | --- | --- | --- | --- | --- | --- | --- |
| 1 | Angermeyer et al., 2015^1^ | Depr. | -0.05 | -0.12 | 0.02 | 9.11% |  |
| 2 | Angermeyer et al., 2015^1^ | Schiz. | -0.05 | -0.12 | 0.02 | 9.11% |  |
| 3 | Makowski et al., 2016a^1^ | Depr. | 0.06 | 0.01 | 0.12 | 9.96% |  |
| 4 | Makowski et al., 2016a^1^ | Schiz. | -0.06 | -0.11 | -0.01 | 9.98% |  |
| 5 | Schomerus et al., 2013^1^ | Depr. | -0.07 | -0.13 | -0.01 | 9.80% |  |
| 6 | Schomerus et al., 2013^1^ | Schiz. | -0.12 | -0.18 | -0.06 | 9.81% |  |
| 7 | Schomerus et al., 2013^1^ | Alc. | -0.07 | -0.13 | -0.01 | 9.73% |  |
| 8 | Speerforck et al., 2019^1^ | ADHD | -0.01 | -0.10 | 0.08 | 8.01% |  |
| 9 | Thibodeau, 2017^1^ | Schiz. | -0.18 | -0.36 | 0.02 | 3.62% |  |
| 10 | Thibodeau & Peterson, 2018^2^ | Schiz. | -0.14 | -0.30 | 0.03 | 4.34% |  |
| 11 | Thibodeau et al., 2018^2^ | Schiz. | -0.12 | -0.53 | 0.33 | 0.96% |  |
| 12 | Thibodeau, 2019^1^ | Depr. | -0.03 | -0.16 | 0.10 | 5.76% |  |
| 13 | Wiesjahn et al., 2016^1^ | Schiz. | -0.20 | -0.25 | -0.14 | 9.81% |  |
|  |  |  | **-0.07** | **-0.11** | **-0.03** |  |  |

Annotations: Population: ^1^ = General population, ^2^ = Undergraduates; Disorder: Depr = Depression, Schiz.= Schizophrenia, Alc = Alcoholism, ADHD = Attention Deficit/Hyperactivity Disorder, OCD = Obsessive-compulsive disorder); r = correlation coefficient; L CI/ U CI = lower and upper limit of confidence interval. Weight: Study weight. Forest Plot: single study effect-sizes and combined effect-size with CI. Size of point reflects study weight.

Table S4.3. Meta-analysis and forest plot of the association of continuum beliefs and anger (single study effect-sizes and combined effect-size).

|  | **Study** | **Disorder** | **r** | **L CI** | **U CI** | **Weight** |  |
| --- | --- | --- | --- | --- | --- | --- | --- |
| 1 | Angermeyer et al., 2015^1^ | Depr. | -0.03 | -0.10 | 0.04 | 10.07% |  |
| 2 | Angermeyer et al., 2015^1^ | Schiz. | 0.16 | 0.09 | 0.23 | 10.07% |  |
| 3 | Makowski et al., 2016a^1^ | Depr. | 0.02 | -0.03 | 0.07 | 10.96% |  |
| 4 | Makowski et al., 2016a^1^ | Schiz. | 0.03 | -0.02 | 0.08 | 10.99% |  |
| 5 | Schomerus et al., 2013^1^ | Depr. | 0.12 | 0.06 | 0.18 | 10.79% |  |
| 6 | Schomerus et al., 2013^1^ | Schiz. | 0.15 | 0.09 | 0.21 | 10.81% |  |
| 7 | Schomerus et al., 2013^1^ | Alc. | 0.10 | 0.04 | 0.16 | 10.73% | 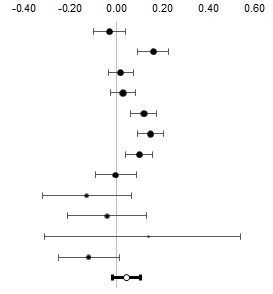 |
| 8 | Speerforck et al., 2019^1^ | ADHD | 0.00 | -0.09 | 0.09 | 8.92% |  |
| 9 | Thibodeau, 2017^1^ | Schiz. | -0.13 | -0.32 | 0.07 | 4.13% |  |
| 10 | Thibodeau & Peterson, 2018^2^ | Schiz. | -0.04 | -0.21 | 0.13 | 4.93% |  |
| 11 | Thibodeau et al., 2018^2^ | Schiz. | 0.14 | -0.31 | 0.54 | 1.12% |  |
| 12 | Thibodeau, 2019^1^ | Depr. | -0.12 | -0.25 | 0.01 | 6.49% |  |
|  |  |  | **0.05** | **-0.01** | **0.10** |  |  |

Annotations: Population: ^1^ = General population, ^2^ = Undergraduates; Disorder: Depr = Depression, Schiz.= Schizophrenia, Alc = Alcoholism, ADHD = Attention Deficit/Hyperactivity Disorder, OCD = Obsessive-compulsive disorder); r = correlation coefficient; L CI/ U CI = lower and upper limit of confidence interval. Weight: Study weight. Forest Plot: single study effect-sizes and combined effect-size with CI. Size of point reflects study weight.

Table S4.4. Meta-analysis and forest plot of the association of continuum beliefs and dangerousness (single study effect-sizes and combined effect-size).

|  | **Study** | **Disorder** | ***r*** | **L CI** | **U CI** | **Weight** |  |
| --- | --- | --- | --- | --- | --- | --- | --- |
| 1 | Makowski et al., 2016a^1^ | Depr. | 0.02 | -0.04 | 0.07 | 16.00% |  |
| 2 | Makowski et al., 2016a^1^ | Schiz. | 0.00 | -0.06 | 0.05 | 16.02% |  |
| 3 | Schlier et al., 2016^1^ | Schiz. | -0.22 | -0.32 | -0.11 | 14.23% |  |
| 4 | Thibodeau, 2017^1^ | Schiz. | -0.04 | -0.23 | 0.16 | 10.63% |  |
| 5 | Thibodeau & Peterson, 2018^2^ | Schiz. | -0.19 | -0.35 | -0.02 | 11.66% |  |
| 6 | Thibodeau et al., 2018^2^ | Schiz. | -0.08 | -0.50 | 0.37 | 4.33% | 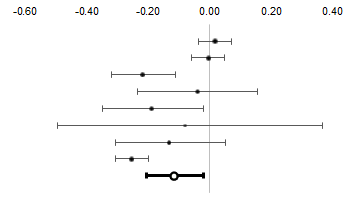 |
| 7 | Wiesjahn et al., 2014^1^ | Schiz. | -0.13 | -0.31 | 0.05 | 11.19% |  |
| 8 | Wiesjahn et al., 2016^1^ | Schiz. | -0.25 | -0.31 | -0.20 | 15.93% |  |
|  |  |  | **-0.12** | **-0.21** | **-0.02** |  |  |

Annotations: Population: ^1^ = General population, ^2^ = Undergraduates; Disorder: Depr = Depression, Schiz.= Schizophrenia); r = correlation coefficient; L CI/ U CI = lower and upper limit of confidence interval. Weight: Study weight. Forest Plot: single study effect-sizes and combined effect-size with CI. Size of point reflects study weight.

Table S4.5. Meta-analysis and forest plot of the association of continuum beliefs and unpredictability (single study effect-sizes and combined effect-size).

|  | **Study** | **Disorder** | ***r*** | **L CI** | **U CI** | **Weight** |  |
| --- | --- | --- | --- | --- | --- | --- | --- |
| 1 | Makowski et al., 2016a^1^ | Depr. | 0.03 | -0.03 | 0.08 | 13.06% |  |
| 2 | Makowski et al., 2016a^1^ | Schiz. | -0.02 | -0.07 | 0.04 | 13.06% |  |
| 3 | Schomerus et al., 2016^1^ | Depr., Schiz. | -0.30 | -0.34 | -0.26 | 13.13% |  |
| 4 | Schlier et al., 2016^1^ | Schiz. | -0.24 | -0.34 | -0.13 | 12.11% | 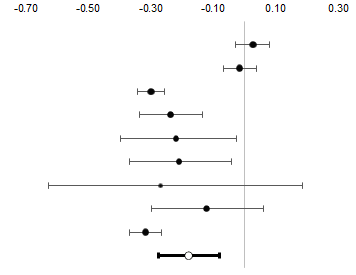 |
| 5 | Thibodeau, 2017^1^ | Schiz. | -0.22 | -0.40 | -0.03 | 9.91% |  |
| 6 | Thibodeau & Peterson, 2018^2^ | Schiz. | -0.21 | -0.37 | -0.04 | 10.58% |  |
| 7 | Thibodeau et al., 2018^2^ | Schiz. | -0.27 | -0.63 | 0.18 | 4.85% |  |
| 8 | Wiesjahn et al., 2014^1^ | Schiz. | -0.12 | -0.30 | 0.06 | 10.28% |  |
| 9 | Wiesjahn et al., 2016^1^ | Schiz. | -0.32 | -0.37 | -0.26 | 13.02% |  |
|  |  |  | **-0.18** | **-0.28** | **-0.08** |  |  |

Annotations: Population: ^1^ = General population, ^2^ = Undergraduates; Disorder: Depr = Depression, Schiz.= Schizophrenia); r = correlation coefficient; L CI/ U CI = lower and upper limit of confidence interval. Weight: Study weight. Forest Plot: single study effect-sizes and combined effect-size with CI. Size of point reflects study weight.

Table S4.6. Meta-analysis and forest plot of the association of continuum beliefs and responsibility (single study effect-sizes and combined effect-size).

|  | **Study** | **Disorder** | ***r*** | **L CI** | **U CI** | **Weight** |  |
| --- | --- | --- | --- | --- | --- | --- | --- |
| 1 | Schomerus et al., 2016^1^ | Depr., Schiz. | 0.06 | 0.01 | 0.11 | 30.12% |  |
| 2 | Schlier et al., 2016^1^ | Schiz. | -0.09 | -0.20 | 0.02 | 24.02% |  |
| 3 | Wiesjahn et al., 2014^1^ | Schiz. | -0.11 | -0.28 | 0.07 | 16.49% |  |
| 4 | Wiesjahn et al., 2016^1^ | Schiz. | -0.10 | -0.16 | -0.04 | 29.37% | **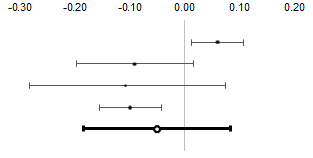** |
|  |  |  | **-0.05** | **-0.18** | **0.08** |  |  |

Annotations: Population: ^1^ = General population, ^2^ = Undergraduates; Disorder: Depr = Depression, Schiz.= Schizophrenia); r = correlation coefficient; L CI/ U CI = lower and upper limit of confidence interval. Weight: Study weight. Forest Plot: single study effect-sizes and combined effect-size with CI. Size of point reflects study weight
