# Supplementary material for: Continuum beliefs and mental illness stigma: a systematic review and meta-analysis of correlation and intervention studies
Source: Psychol Med. 2021 Apr 8;51(5):716–26. doi: 10.1017/S0033291721000854 (PMC8108391; doi:10.1017/S0033291721000854)
Supplement: Supplementary file 1 [file S0033291721000854sup.zip › S0033291721000854sup005.docx]

# Appendix S5: Meta-Analyses with separate effect-sizes

Table S5.1: Separate meta-analyses for the association of continuum beliefs and social distance.

|  |  |  | | | | CI | | | Heterogeneity | | | |
| --- | --- | --- | --- | --- | --- | --- | --- | --- | --- | --- | --- | --- |
| Outcome | | *k* | *r* | *r_p_* | *p* | L | U | *I²* | | *Q* | *df* | *p* |
| Overall combined r | | 10 | **-0.20** |  | < 0.001 | -0.27 | -0.13 | 86.02 | | 64.37 | 9 | < 0.001 |
|  | Schizophrenia | 8 | **-0.21** |  | < 0.001 | -0.30 | -0.13 | 86.73 | | 52.74 | 7 | < 0.001 |
|  | Subgroup 2 | 7 | **-0.24** |  | < 0.001 | -0.31 | -0.17 | 61.92 | | 18.38 | 6 | 0.01 |
|  | Subgroup 2  (only Thibodeau studies) | 4 | **-0.19** |  | < 0.001 | -0.29 | -0.08 | 0.00 | | 1.58 | 3 | 0.663 |
|  | Subgroup 2  (without undergraduates) |  | -0.25 |  | < 0.001 | 0.33 | -0.17 | 68.00 | | 15.63 | 5 | < 0.001 |
| *Overall combined ß* | | 11 |  | **-0.09** | < 0.001 | -0.12 | -0.05 | 43.67 | | 17.75 | 10 | 0.059 |
|  | depression | 3 |  | 0.07 | 0.121 | -0.25 | 0.12 | 74.45 | | 7.83 | 2 | 0.020 |
| schizophrenia | 3 |  | **-0.10** | < 0.001 | -0.13 | -0.08 | 0.00 | | 0.22 | 2 | 0.896 |  |

Annotations: Outcome: overall results and subgroup analyses: type of disorder (depression, schizophrenia), methods (subgroup 1 = one-item measure of Schomerus, regression models; subgroup 2 = Thibodeau’s measure, correlation models). k = number of effect-sizes. r = combined correlation coefficient. p = two-tailed p-value of combined r. L CI/ U CI = Lower and upper limit of confidence interval. Heterogeneity = I², Q-value with df and p-value. Bold = Confidence interval does not contain zero.

Table S5.2. Separate meta-analyses for the association of continuum beliefs and pro-social-reactions.

|  |  |  | | | | | | | | CI | | | Heterogeneity | | | | | | |  |
| --- | --- | --- | --- | --- | --- | --- | --- | --- | --- | --- | --- | --- | --- | --- | --- | --- | --- | --- | --- | --- |
| Outcome | | *k* | *r* | *rp* | | | *p* | | L | | U | | *I²* | | *Q* | | *df* | | *p* |  |
| Overall combined r | | 6 | -0.01 | |  | 0.894 | | -0.26 | | | | 0.24 | | 85.50 | | 34.47 | | 5 | < 0.001 | |
|  | Schizophrenia | 4 | -0.17 | |  | 0.234 | | -0.57 | | | | 0.29 | | 88.33 | | 25.72 | | 3 | < 0.001 | |
|  | Subgroup 2  (only Thibodeau studies) | 4 | -0.16 | |  | 0.343 | | -0.61 | | | | 0.36 | | 88.58 | | 26.26 | | 3 | < 0.001 | |
|  | Subgroup  (without undergraduates) | 4 | 0.10 | |  | 0.003 | | -0.01 | | | | 0.20 | | 44.49 | | 5.4 | | 3 | 0.144 | |
| *Overall combined ß* | | 6 |  | | **0.10** | | < 0.001 | 0.09 | | | | 0.12 | | 00.00 | | 0.72 | | 5 | 0.982 | |
|  | Subgroup 1  (adding 2 studies) | 9 |  | | **0.10** | | < 0.001 | 0.08 | | | | 0.11 | | 00.00 | | 2.60 | | 8 | 0.957 | |

Annotations: Outcome: overall results and subgroup analyses: type of disorder (depression, schizophrenia), methods (subgroup 1 = one-item measure of Schomerus, regression models; subgroup 2 = Thibodeau’s measure, correlation models). k = number of effect-sizes. r = combined correlation coefficient. p = two-tailed p-value of combined r. L CI/ U CI = Lower and upper limit of confidence interval. Heterogeneity = I², Q-value with df and p-value. Bold = Confidence interval does not contain zero.

Table S5.3. Meta-Analysis of continuum beliefs and desire of **social distance** with **correlation coefficient** as effect-size.

|  | **Study** | **Disorder** | ***r*** | ***L CI*** | ***U CI*** | **Weight** | 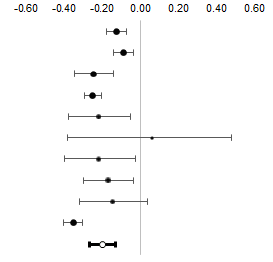 |
| --- | --- | --- | --- | --- | --- | --- | --- |
| 1 | Makowski et al., 2016^1^ | Depr. | -0.13 | -0.18 | -0.07 | 13.24% |  |
| 2 | Makowski et al., 2016^1^ | Schiz. | -0.09 | -0.14 | -0.04 | 13.25% |  |
| 3 | Schlier et al., 2016^1^ | Schiz. | -0.25 | -0.34 | -0.14 | 11.07% |  |
| 4 | Schomerus et al., 2016^1^ | Depr., Schiz. | -0.25 | -0.29 | -0.20 | 13.43% |  |
| 5 | Thibodeau et al., 2018a^2^ | Schiz. | -0.22 | -0.38 | -0.05 | 8,29% |  |
| 6 | Thibodeau et al., 2018b^2^ | Schiz. | 0.06 | -0.38 | 0.48 | 2.49% |  |
| 7 | Thibodeau et al., 2017^1^ | Schiz. | -0.22 | -0.40 | -0.03 | 7.32% |  |
| 8 | Thibodeau et al., 2019^1^ | Depr. | -0.17 | -0.30 | -0.04 | 9,90% |  |
| 9 | Wiesjahn et al., 2014^1^ | Schiz. | -0.15 | -0.32 | 0.04 | 7.87% |  |
| 10 | Wiesjahn et al., 2016^1^ | Schiz. | -0.35 | -0.40 | -0.30 | 13.14% |  |
|  |  |  | **-0.20** | **-0.27** | **-0.13** |  |  |

Annotations: Population: ^1^ = General population, ^2^ = Undergraduates; Disorder: Depr = Depression, Schiz.= Schizophrenia, Alc = Alcoholism); r = correlation coefficient; L CI/ U CI = lower and upper limit of confidence interval. Weight: Study weight. Forest Plot: single study effect-sizes and combined effect-size with CI. Size of point reflects study weight.

Table S5.4. Meta-Analysis for the association of continuum beliefs and desire of **social distance** with **partial correlation coefficient** as effect-size.

|  | **Study** | **Disorder** | ***r_p_*** | ***L CI*** | ***U CI*** | **Weight** | 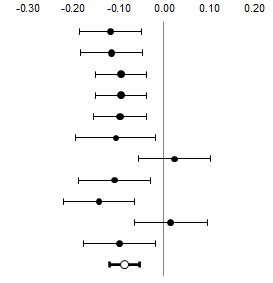 | |
| --- | --- | --- | --- | --- | --- | --- | --- | --- |
| 1 | Angermeyer et al., 2015^1^ | Depr. | -0.12 | -0.19 | -0.05 | 9,47% |  |  |
| 2 | Angermeyer et al., 2015^1^ | Schiz. | -0.12 | -0.18 | -0.05 | 9.47% |  |  |
| 3 | Schomerus et al., 2013^1^ | Depr. | -0.09 | -0.15 | -0.04 | 11.50% |  |  |
| 4 | Schomerus et al., 2013^1^ | Schiz. | -0.10 | -0.15 | -0.04 | 11.54% |  |  |
| 5 | Schomerus et al., 2013^1^ | Alc. | -0.10 | -0.16 | -0.04 | 11.29% |  |  |
| 6 | Speerforck et al., 2019^1^ | ADHD | -0.11 | -0.19 | -0.02 | 7.00% |  |  |
| 7 | Subramaniam et al., 2017^1^ | Depr. | 0.02 | -0.06 | 0.10 | 7.92% |  |  |
| 8 | Subramaniam et al., 2017^1^ | Schiz. | -0.11 | -0.19 | -0.03 | 7.93% |  |  |
| 9 | Subramaniam et al., 2017^1^ | Alc. | -0.14 | -0,22 | -0.06 | 8.08% |  |  |
| 10 | Subramaniam et al., 2017^1^ | Dem. | 0.01 | -0.07 | 0.10 | 7.82% |  |  |
| 11 | Subramaniam et al., 2017^1^ | OCD | -0.10 | -0.18 | -0.02 | 7.99% |  |  |
|  |  |  | **-0.09** | **-0.12** | **-0.05** |  |  | |

Annotations: Population: ^1^ = General population; Disorder: Depr = Depression, Schiz.= Schizophrenia, Alc = Alcoholism, ADHD = Attention Deficit/Hyperactivity Disorder, Dement = Dementia, OCD = Obsessive-compulsive disorder r_p_ = partial correlation coefficient; L CI/ U CI = lower and upper limit of confidence interval. Weight: Study weight. Forest Plot: single study effect-sizes and combined effect-size with CI. Size of point reflects study weight.

Table S5.5. Meta-Analysis of continuum beliefs and **pro-social reactions** with **correlation coefficient** as effect-size.

|  | **Study** | **Disorder** | ***r*** | **L CI** | **U CI** | **Weight** |  |
| --- | --- | --- | --- | --- | --- | --- | --- |
| 1 | Makowski et al., 2016^1^ | Depr | 0,12 | 0,06 | 0,17 | 22,86% |  |
| 2 | Makowski et al., 2016^1^ | Schiz | 0,07 | 0,02 | 0,13 | 22,88% |  |
| 3 | Thibodeau et al., 2018a^2^ | Schiz | -0,27 | -0,42 | -0,10 | 15,95% |  |
| 4 | Thibodeau et al., 2018b^2^ | Schiz | -0,59 | -0,81 | -0,21 | 5,54% | *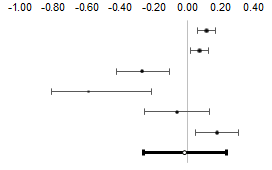* |
| 5 | Thibodeau et al., 2017^1^ | Schiz | -0,06 | -0,25 | 0,14 | 14,40% |  |
| 6 | Thibodeau et al., 2019^1^ | Depr | 0,18 | 0,05 | 0,31 | 18,37% |  |
|  |  |  | **-0.01** | **-0.26** | **0.24** |  |  |

*Annotations: Population:* ^1^*= General population, 2 = Undergraduates; Disorder: Depr = Depression, Schiz.= Schizophrenia); r = correlation coefficient; L CI/ U CI = lower and upper limit of confidence interval. Weight: Study weight. Forest Plot: single study effect-sizes and combined effect-size with CI. Size of point reflects study weight.*

Table S5.6. Meta-Analysis of continuum beliefs and **pro-social reactions** with **partial correlation** as effect-size.

|  | Study | Disorder | *r_p_* | *L CI* | *U CI* | Weight | 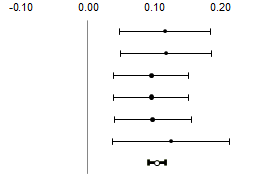 |
| --- | --- | --- | --- | --- | --- | --- | --- |
| 1 | Angermeyer et al., 2015^1^ | Depr | 0.12 | 0.05 | 0.18 | 14.33% |  |
| 2 | Angermeyer et al., 2015^1^ | Schiz | 0.12 | 0.05 | 0.19 | 14.34% |  |
| 3 | Schomerus et al., 2013^1^ | Depr | 0.10 | 0.04 | 0.15 | 21.04% |  |
| 4 | Schomerus et al., 2013^1^ | Schiz | 0.10 | 0.04 | 0.15 | 21.21% |  |
| 5 | Schomerus et al., 2013^1^ | Alc | 0.10 | 0.04 | 0.16 | 20.24% |  |
| 6 | Speerforck et al., 2019^1^ | ADHD | 0.12 | 0.04 | 0.21 | 8.84% |  |
|  |  |  | **0.10** | **0.09** | **0.12** |  |  |

*Annotations: Population:* ^1^*= General population; Disorder: Depr = Depression, Schiz.= Schizophrenia, ADHD = Attention Deficit/Hyperactivity Disorder; r_p_ = partial correlation coefficient; L CI/ U CI = lower and upper limit of confidence interval. Weight: Study weight. Forest Plot: single study effect-sizes and combined effect-size with CI. Size of point reflects study weight.*
